# Novel Perovskite Oxide Hybrid Nanofibers Embedded with Nanocatalysts for Highly Efficient and Durable Electrodes in Direct CO_2_ Electrolysis

**DOI:** 10.1007/s40820-023-01298-w

**Published:** 2024-01-22

**Authors:** Akromjon Akhmadjonov, Kyung Taek Bae, Kang Taek Lee

**Affiliations:** 1 Department of Mechanical Engineering, KAIST, Daejeon, 34141 Republic of Korea; 2 KAIST Graduate School of Green Growth and Sustainability, Daejeon, 34141 Republic of Korea

**Keywords:** Nanofibers, Fuel electrodes, Digital twinning, CO_2_ reduction reaction, Solid oxide electrolysis cells

## Abstract

The unique characteristics of nanofibers in rational electrode design enable effective utilization and maximizing material properties for achieving highly efficient and sustainable CO_2_ reduction reactions (CO_2_RRs) in solid oxide electrolysis cells (SOECs). However, practical application of nanofiber-based electrodes faces challenges in establishing sufficient interfacial contact and adhesion with the dense electrolyte. To tackle this challenge, a novel hybrid nanofiber electrode, La_0.6_Sr_0.4_Co_0.15_Fe_0.8_Pd_0.05_O_3−*δ*_ (H-LSCFP), is developed by strategically incorporating low aspect ratio crushed LSCFP nanofibers into the excess porous interspace of a high aspect ratio LSCFP nanofiber framework synthesized via electrospinning technique. After consecutive treatment in 100% H_2_ and CO_2_ at 700 °C, LSCFP nanofibers form a perovskite phase with in situ exsolved Co metal nanocatalysts and a high concentration of oxygen species on the surface, enhancing CO_2_ adsorption. The SOEC with the H-LSCFP electrode yielded an outstanding current density of 2.2 A cm^−2^ in CO_2_ at 800 °C and 1.5 V, setting a new benchmark among reported nanofiber-based electrodes. Digital twinning of the H-LSCFP reveals improved contact adhesion and increased reaction sites for CO_2_RR. The present work demonstrates a highly catalytically active and robust nanofiber-based fuel electrode with a hybrid structure, paving the way for further advancements and nanofiber applications in CO_2_-SOECs.

**Supplementary Information:**

The online version contains supplementary material available at 10.1007/s40820-023-01298-w.

## Highlights

- The novel hybrid structured nanofiber electrode, incorporating crushed nanofibers into the nanofiber network, is designed to effectively resolve the issue of insufficient interfacial bonding of porous nanofiber electrodes on solid electrolyte interfaces.
- The hybrid nanofibers are covered with in situ exsolved metal nanocatalysts to enhance CO_2_ reduction reaction rate.
- Electrochemical and microstructure 3D reconstruction analyses confirmed the hybrid structure’s efficiency in improving the contact area at the porous-solid interface, leading to an increase in reaction sites.

**Supplementary Information:**

The online version contains supplementary material available at 10.1007/s40820-023-01298-w.

## Graphical abstract

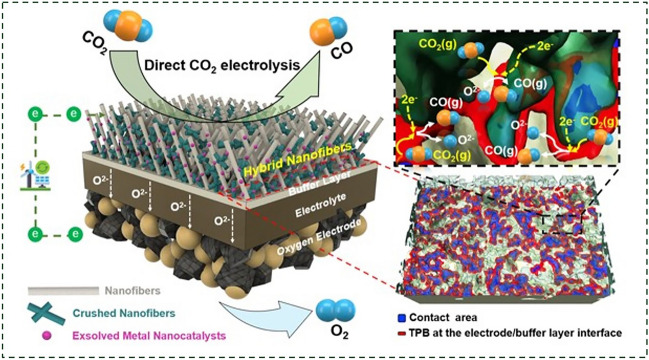

## Introduction

The persistent global challenge of CO_2_ emissions has spurred intensified efforts to accelerate the wide implementation of CO_2_ capture, utilization, and storage (CCUS) strategies [1]. In this context, high-temperature solid oxide electrolysis cells (HT-SOECs) have emerged as vital components in the CCUS ecosystem, enabling the efficient conversion of notoriously inert CO_2_ molecules into valuable chemical compounds using surplus renewable electricity. [2, 3]. HT-SOECs consist of fuel and oxygen electrodes, separated by an oxygen ion conductive electrolyte [4]. The fuel electrode plays a central role in electrochemical reduction of CO_2_ into CO and O^2−^ [5]. The generated O^2−^ migrates through the solid electrolyte and converts into O_2_ in the oxygen electrode [6], while the CO product can be further upgraded into high-energy–density, long-chain hydrocarbons through the Fischer–Tropsch process [7].

The utilization of conventional catalytically active Ni-based cermets as fuel electrode materials for CO_2_-SOECs has been limited due to stability issues encountered in carbon-rich environments during prolonged operation, such as re-oxidation or carbon deposition [8–10]. Consequently, the research focus has shifted toward the development of fuel electrode materials that possess not only high catalytic activity but also redox stability and carbon tolerance in a CO_2_ environment [11]. Several perovskite-based mixed ionic and electronic conducting (MIEC) materials, such as lanthanum strontium cobalt ferrite (La_1−*x*_Sr_*x*_Co_1−*y*_Fe_*y*_O_3−*δ*_, LSCF) and Sr_2_Fe_1.5_Mo_0.5_O_6−*δ·*_ -based, have been reported to fulfill these criteria [12, 13]. To enhance the catalytic activity of LSCF, researchers have employed surface modification techniques, such as in situ exsolution, wherein catalytically active metal nanoparticles are decorated on the perovskite surface [14, 15]. Recently, Lee et al*.* developed a high-performance and durable Pd-doped LSCF (LSCFP) fuel electrode material by introducing 5 mol% Pd doping into the B-site of the LSCF perovskite, which concurrently promoted phase transition to the RP phase under the reducing condition at lower temperature and facilitated the in situ exsolution of catalytically active metal nanocatalysts on the surface [16].

In addition, the rational design of electrode microstructure plays a crucial role in effectively utilizing and enhancing a material’s properties, including catalytic activity [17, 18]. Extensive research has, therefore, focused on designing nanostructured electrodes [19], with particular interest in one-dimensional nanomaterials such as nanofibers due to their high porosity for facile gas diffusion, pathways for ionic and electronic conductivity, and large surface area for an increased number of reaction sites [20, 21]. For instance, Liu et al*.* reported the fabrication of the nanofiber oxygen electrode for solid oxide fuel cell (SOFC) composed of LSCF nanofibers that exhibited a maximum power density (MPD) of 0.62 W cm^−2^ and demonstrated excellent durability for 450 h at 600 °C. Their high performance was attributed to the unique architecture of the long and thin fibers, facilitating efficient mass and charge transport [22]. More recently, Jung et al*.* demonstrated a remarkable reduction in polarization resistance and high stability of nanofibers incorporated in a nanocomposite electrode during direct hydrocarbon-fueled operation [23].

Despite the numerous structural advantages offered by nanofiber-based electrodes, their practical application has been limited owing to their insufficient interfacial contact and adhesion on the dense electrolyte [24]. To overcome this hurdle, some researchers have reported solutions involving an interlayer deposition using conventional powder materials between the nanofiber electrode and electrolyte [24, 25]. Although the insertion of the powder layer increases the adhesion of the nanofiber electrode, it introduces additional resistance to ionic conduction [10], and the actual electrochemical reaction at the electrode occurs in the powder interlayer rather than the nanofibers, largely nullifying the effectiveness of using nanofibers. On the other hand, Wei et al*.* demonstrated a robust Li_1.18_Co_0.15_Ni_0.15_Mn_0.52_O_2_ electrode with long-range electron pathways for Li-ion batteries by bridging isolated electrode particle regions with incorporated vapor-grown carbon fibers. This study suggests that combining high aspect ratio nanofibers with materials with lower aspect ratio synergistically enhances connectivity within the bulk electrode and facilitates charge transfer [26].

Inspired by these previous studies, we have designed a novel structure for the nanofiber electrode by strategically incorporating crushed nanofibers (thus, with lower aspect ratio) into the excess porous interspace of the nanofiber matrix. This hybrid structured nanofiber electrode effectively creates additional surfaces at the two-phase interface and within the bulk electrode without agglomeration, improving the contact adhesion and increasing the number of reaction sites available at the fuel electrode/electrolyte interface for CO_2_-SOECs.

In this study, we prepared a hybrid nanofiber LSCFP (referred to as H-LSCFP) fuel electrode by incorporating crushed LSCFP nanofibers into the original nanofiber matrix synthesized using electrospinning technology. The crystallographic structure, composition, and surface chemistry of the H-LSCFP were systematically analyzed, and the CO_2_ electrolysis performance of SOECs with the H-LSCFP electrode was evaluated. Additionally, the correlation between microstructural characteristics of the fuel electrode and its CO_2_ electrolysis performance was elucidated through the digital twinning using a state-of-the art 3D reconstruction technique.

## Experimental Section

### Electrode Preparation

Stoichiometric amounts of La(NO_3_)_3_·6H_2_O (99.9%, Alfa Aesar), Sr(NO_3_)_2_ (99.99%, Sigma-Aldrich), Co(NO_3_)_2_·6H_2_O (99.99%, Alfa Aesar), and Fe(NO_3_)_3·_9H_2_O (99.99%, Alfa Aesar) and Pd(OCOCH_3_)_2_ (99.999%, Sigma-Aldrich) were dissolved in a Dimethylformamide (Alfa Aesar) solvent. Subsequently, Polyvinylpyrrolidone (Sigma-Aldrich) polymer was added to the precursor solution and stirred until a viscous LSCFP polymer precursor solution was obtained. The solution was then pumped through a plastic syringe using a 25-gauge plastic needle nozzle at a feed rate of 0.25 mL h^−1^. A high voltage of 17.5 kV was applied to the needle, while the collector was grounded at 0 V, and the distance between the needle and collector was fixed at approximately 17–18 cm. The resulting as-electrospun nanofibers were calcined in air at 1000 °C for 2 h, at a heating rate of 2 °C min^−1^ to obtain the fiber ash. For the F-LSCFP electrode ink, the LSCFP nanofiber ash was ultrasonically dispersed and mixed with a binder (441 ESL, Electro Science) at a ratio of 0.15 to 0.2. The H-LSCFP electrode ink followed the same procedure but also included crushed LSCFP nanofibers. A comprehensive depiction of the electrospinning process employed for the synthesis of LSCFP nanofibers is shown in Fig. S1, while a step-by-step description of the H-LSCFP electrode fabrication procedure is illustrated in Fig. S2.

### Characterization

X-ray diffraction (XRD) patterns of the LSCFP nanofibers were analyzed over a 2*θ* range of 20°–80° using a high-resolution X-ray diffractometer (Smartlab, Rigaku) with CuK*α* radiation (*λ* = 1.54 Å). The crystal structures were refined using SmartLab Studio II software package (Rigaku). The microstructure of the nanofibers and cell components was observed using field-emission scanning electron microscopy (FE-SEM, S-8230, Hitachi). Morphology and compositional properties of the LSCFP nanofibers were examined using high-resolution transmission electron microscopy (HR-TEM, Talos F200X, FEI) equipped with energy-dispersive X-ray (EDX) spectroscopy (Bruker). Surface oxidation states of the LSCFP nanofibers were analyzed by X-ray photoelectron spectroscopy (XPS, Nexsa G2, Thermo Fisher) with the CASA XPS software package. The CO_2_-temperature programmed desorption (CO_2_-TPD) was performed using an Autochem II 2920 (Micromeritics) equipped with a thermal conductivity detector. Measurements were conducted in a U-type quartz reactor using 0.2 g of sample. The sample was pre-treated in 50 sccm He at 400 °C for 1 h. Then, the CO_2_ adsorption experiment was carried out in CO_2_ of 50 sccm at 900 °C for 1 h. After cooling the sample to room temperature, the TPD curves were measured in 50 sccm He from room temperature to 900 °C.

### Cell Fabrication for CO_2_ Electrolysis of SOEC

Symmetric cells were prepared using La_0.8_Sr_0.2_Ga_0.8_Mg_0.2_O_3−*δ*_ (LSGM, Kceracell) pellets uniaxially pressed at 40 MPa and sintered at 1450 °C for 5 h. The prepared electrode ink was coated on both sides of the LSGM pellet, resulting in a symmetric cell configuration of electrode||electrolyte||electrode. For the fabrication of electrolyte-supported single CO_2−_SOECs, the LSGM electrolyte was prepared by a tape-casting method [27]. First, La_0.8_Sr_0.2_Ga_0.8_Mg_0.2_O_3−*δ*_ (LSGM, Kceracell) powders were ram-mixed with a methyl ethyl ketone (MEK, Samchun), ethanol (Supelco), triton (Alfa Aesar), and a polyethylene glycol (PEG300, Samchun Chemicals) mixture. Subsequently, polyvinyl butyral (BUTVAR B-79) and dibutyl phthalate (DBP, Junsei) were sequentially introduced as binders and plasticizers and thoroughly mixed. After degassing for 1 min under vacuum while mixing, the LSGM slurry was cast onto a polyester Mylar film using a doctor blade on a bench-top tape caster (Hantech, Co., LTD) and dried for 24 h in ambient air at room temperature. The obtained LSGM tape was then punched into 25-mm circular disks and then sintered at 1450 °C for 5 h. The resulting sintered disk was polished to a thickness of approximately 200–220 μm. To prevent the formation of unwanted secondary phases, a LDC (Kceracell) buffer layer was introduced between the fuel electrode and electrolyte. The oxygen electrode consisted of a composite of LSCF (Fuelcellmaterials) and GDC (Rhodia) in a ratio of 50:50 wt%. The oxygen and fuel electrode slurries were applied to the LSGM electrolyte by blade-coating and sintered at 1100 °C for 3 h, at a heating rate of 3 °C min^−1^. The active area of the electrodes was approximately 0.5 cm^2^.

### Electrochemical Measurements

The electrode resistance of the LSGM electrolyte-supported symmetric cell was evaluated in the temperature range of 700–850 °C using a potentiostat (VMP-300, Bio-Logic). The measurements were carried out with a 100 mV AC amplitude signal at an applied potential of 1.5 V in 100% CO_2_ of 50 sccm. The frequency range for the impedance analysis was set from 0.1 Hz to 1 MHz. The distribution of relaxation time (DRT) analysis of the obtained impedance data was performed using DRT Tools developed by Ciucci et al*.* [28]. The current–voltage (*I*–*V*) characteristics of the SOECs with H-LSCFP and F-LSCFP fuel electrodes were measured in a custom-built airtight single-cell measurement system. The edge between the cell and alumina reactor was made gas-tight by sealing with Ceramabond 517 (Aremco). The electrochemical performance of CO_2_-SOECs was evaluated using a potentiostat (VMP-300, Bio-Logic). CO_2_ was supplied to the fuel electrode at a flow rate of 50 sccm, while the oxygen electrode remained exposed to ambient air. In the FC tests, the fuel electrode was supplied with humidified hydrogen (3% H_2_O) at a flow rate of 200 sccm, while the oxygen electrode was exposed to dry air (200 sccm). The off-gas product analysis was conducted using an on-line gas chromatograph (GC-7820, Shimadzu) equipped with a thermal conductivity cell detector (TCD). The rate of gas flow was measured using a flowmeter, and Faradaic efficiency was calculated using the following equation:

1 $${{\text{FE}}}_{{\text{CO}}}=\frac{{n}_{{\text{CO}}, {\text{measured}}}}{{n}_{{\text{CO}},{\text{theoretical}}}}\times 100\%=\frac{{n}_{{\text{CO}}, {\text{measured}}}\times {Z}_{{\text{CO}}}\times F}{I \times t}\times 100\%$$

where ${n}_{{\text{CO}},\mathrm{ measured}}$ (mol s^−1^) represents the measured carbon monoxide (CO) production rate obtained from the exhaust gas of the electrochemical cell (GC data). $I$(A) denotes the current, ${Z}_{{\text{CO}}}$ is the number of electrons involved in CO molecule production, F stands for Faraday’s constant (96,485 C mol^−1^), and $t$ represents time.

### Digital Twin of Electrodes

For the digital twinning of the nanofiber-based electrodes, a focused ion beam (FIB)-SEM dual beam system (ZEISS, Crossbeam 550) was utilized. To enhance the contrast of the SEM images and improve pore recognition within the electrodes, an epoxy resin (EpoVac system, Struers) was vacuum-infiltrated into both samples. The Smart FIB software (Zeiss) was utilized to perform ion beam slicing and SEM imaging, generating images at 32.5 nm intervals along the *z*-axis. To prevent curtaining effects and charging during image acquisition, we coated the region of interest with platinum [27, 29–33]. For the 3D reconstruction of the obtained image series, various processes, including cropping, alignment, and segmentation, were carried out using AVIZO software (Visualization Science Group). The segmentation process was particularly crucial for the quantitative analysis of the nanofiber-based electrodes, enabling the identification of distinct phases. Initially, we applied image de-noising using a non-local means filter [34], a well-established technique renowned for noise reduction, including curtaining effects, while preserving edge intensity. Phase designation was achieved using the watershed segmentation algorithm [35, 36], which calculates voxel gradients, treating them as a 2D or 3D landscape. Based on the segmented data, quantified parameters such as volume fraction, phase connectivity, surface area, and TPB density were calculated from Avizo software. Additionally, we determined the tortuosity factor of each electrode using a MATLAB plug-in called the Tau Factor [37].

## Results and Discussion

### Crystallographic Phase Analysis

**Fig. 1:**
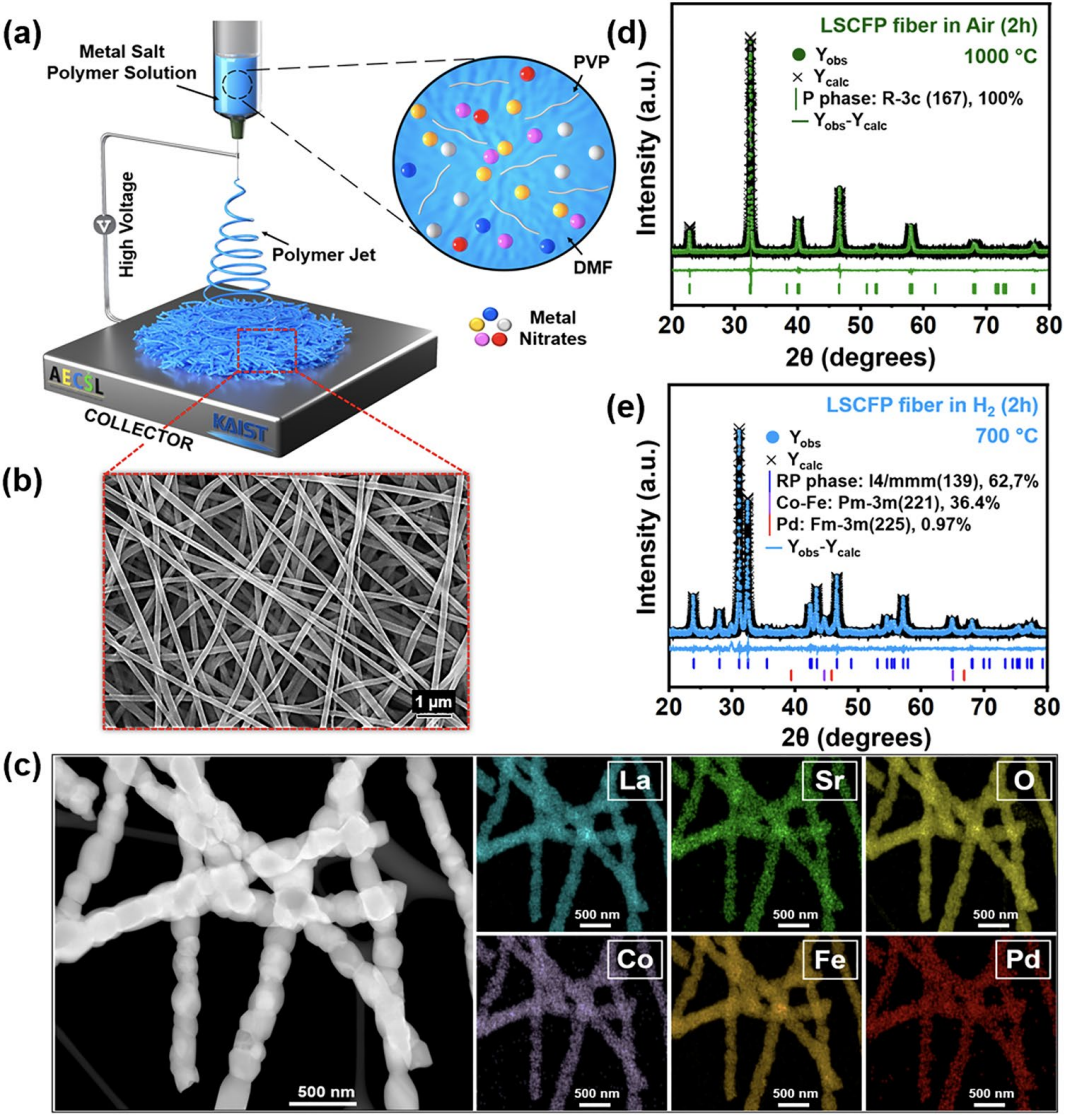
**a** Schematic illustration depicting the synthesizing process of LSCFP nanofibers. **b** SEM image of the as-electrospun LSCFP nanofibers before calcination. **c** TEM–EDX elemental mappings of La (cyan), Sr (green), Co (purple), Fe (dark orange), Pd (red), and O (yellow) for the calcined LSCFP nanofibers. Rietveld XRD refinement of the LSCFP nanofibers **d** calcined in air at 1000 °C for 2 h and **e** LSCFP nanofibers treated in 100% H_2_ of 100 sccm at 700 °C for 2 h. (Colour figure online)

Figure 1a illustrates the electrospinning technique used to synthesize nanofiber mats from LSCFP precursor polymer solution. In Fig. 1b, a magnified SEM image of the nanofiber mats exhibits their porous and interconnected structure. The as-synthesized nanofibers were subsequently calcined in air at 1000 °C for 2 h. Figure 1c depicts a TEM image and the corresponding EDX mapping images of the calcined nanofibers, which confirmed the uniform distribution of all constituent elements (La, Sr, Co, Fe, Pd, and O) within the nanofibers. HR-TEM in Fig. S3 revealed an interplanar distance of 2.74 Å, corresponding to the (110) planes of the perovskite structure. Figure 1d shows the Rietveld refinement analysis of the calcined LSCFP fibers, indicating a successful formation of a perovskite phase (space group *R-3c*, 100 wt%) structure with a space group of R-3c, lattice parameters of *a* = *b* = 5.50 Å, and a lattice volume of 351.86 Å^3^, without any impurities or secondary peaks. The goodness of fit (*χ*^2^) parameter value of 0.94 attested to the validity of the refinement results, indicating a good agreement between the observed and calculated data for the calcined LSCFP nanofibers.

The calcined nanofibers were reduced in 100% H_2_ of 100 sccm at 700 °C for 2 h. Figure 1e presents the Rietveld refinement results, showing the decomposition of the LSCFP nanofibers from the perovskite to the Ruddlesden–Popper (RP) phase of the tetragonal crystal structure (LaSrFeO_4_, space group  *I4/mmm*, 62.7 wt%, *a* = *b* = 3.88 Å, *c* = 12.72 Å), with the metallic phases of the Co–Fe alloy (space group *Pm-3 m*, 36.4 wt%, *a* = *b* = *c* = 2.86 Å) and Pd (space group, *Fm-3 m*, 0.97  wt%, *a* = *b* = *c* = 3.95 Å). These results were consistent with a previous report [16]. The scanning TEM (STEM) image in Fig. S4a reveals the formation of nanoparticles with an average size of ~ 15 nm, exsolved on the parent nanofiber surface. Point analysis of an exsolved nanoparticle revealed a significant presence of Co and Fe in the Co–Fe alloy, with atomic ratios of 28.56% and 46.48%, respectively (Fig. S5). An HR-TEM analysis of the nanoparticle core (Fig. S4h) and particle/support interface (Fig. S4i) yielded interplanar distances of 0.203 and 0.257 nm, respectively, corresponding to the (110) crystal planes of the Co–Fe alloy (Pm-3 m (221)) and (112) planes of the RP structure (*I4*/*mmm* (139)).

**Fig. 2:**
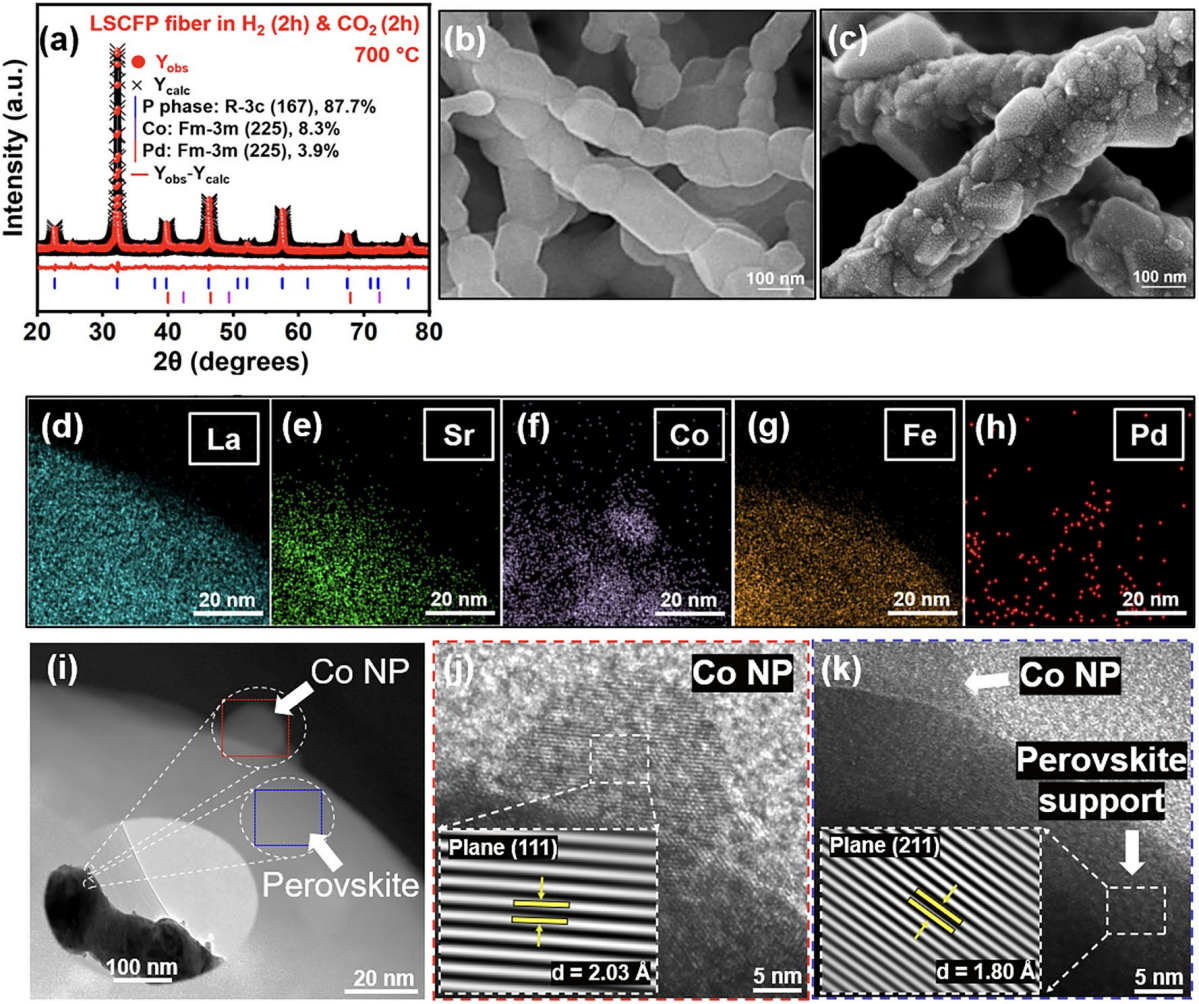
**a** Rietveld XRD refinement of the LSCFP nanofibers consecutively heat treated in 100% H_2_ (100 sccm) and CO_2_ (100 sccm) at 700 °C for 2 h each. SEM comparison images of the **b** pristine and **c** nanofibers consequently treated in 100% H_2_ and CO_2_ at 700 °C. **d–h** TEM–EDX elemental mappings of La (cyan), Sr (green), Co (purple), Fe (dark orange), and Pd (red). **i** Magnified STEM image of exsolved Co nanoparticle with HR-TEM image of **j** the particle and **k** LSCFP perovskite matrix. (Colour figure online)

The reduced LSCFP nanofibers were exposed to 100% CO_2_ of 100 sccm at 700 °C for 2 h. Figure 2a shows the refinement results, which demonstrate that the reduced LSCFP nanofibers reverted back to perovskite (space group *R-3c*, 87.7 wt%, *a* = *b* = 5.54, *c* = 13.58 Å) with a metallic phase of Co (space group *Fm-3 m*, 8.3 wt%, *a* = *b* = *c* = 3.69 Å) and Pd (space group *Fm-3 m*, 3.9 wt%, *a* = *b* = *c* = 3.9 Å) after the 100% CO_2_ treatment. The resulting perovskite structure differed from that of the nanofibers calcined in air, as evidenced by a slight shift in the diffraction peaks to a lower 2*θ* value. Furthermore, unlike the clear surface of the LSCFP nanofibers calcined in air (Fig. 2b), the LSCFP nanofibers consecutively treated in 100% H_2_ and CO_2_ exhibited exsolved nanoparticles on their surfaces (Fig. 2c). The EDX elemental mapping images in Fig. 2d–h show that the exsolved nanoparticles were composed of Co, further confirmed through point analysis (Fig. S6), indicating that Fe phase reincorporated back into the parent perovskite lattice upon CO_2_ treatment. Fe was estimated to redissolve back into the parent perovskite lattice within 30 min when the LSCFP nanofibers were exposed to 100% CO_2_ (Fig. S7). The interplanar spacing of the nanoparticle shown in red rectangle in Fig. 2i was found to be 0.203 nm (Fig. 2j), corresponding to the (111) plane of face-centered cubic (FCC) structured Co, whereas the interplanar distance of the nanofiber oxide shown in the blue rectangle (Fig. 2k) was observed to be 0.18 nm, consistent with the (211) plane of the perovskite structure. Additionally, we observed several cases of localized exsolution of Pd nanoparticles on the LSCFP surface, which also was confirmed through point analysis (Fig. S8). Table 1 provides the corresponding details of the lattice parameters and space groups for each state.

**Table 1:** Lattice parameters derived from Rietveld refinement of LSCFP nanofibers at different states

Material	State	Phase	Crystal system	Space group	a [Å]	b [Å]	c [Å]	a [Å^3^]	*R*_wp_	*R*_p_	*Χ*^2^
LSCFP	Pristine	La_0.6_Sr_0.4_Co_0.2_Fe_0.8_O_3−*δ*_	Trigonal	R-3c (167)	5.50	5.50	13.39	351.86	12.95	8.79	0.94
	Reduced	LaSrFeO_4_	Tetragonal	I4/mmm (139)	3.88	3.88	12.72	192.44			
		Co–Fe	Cubic	Pm-3m (221)	2.86	2.86	2.86	23.54	12.35	8.96	0.95
		Pd	Cubic	Fm-3m (225)	3.95	3.95	3.95	61.94			
	Reduced & CO_2_ treated	(La_0.6_Sr_0.4_)FeO_2.71_	Trigonal	R-3c (167)	5.54	5.54	13.58	362.15			
		Co	Cubic	Fm-3m (225)	3.69	3.69	3.69	50.2	5.3	4.03	1.72
		Pd	Cubic	Fm-3m (225)	3.9	3.9	3.9	59.28			

### Oxidation States of Elements

**Fig. 3:**
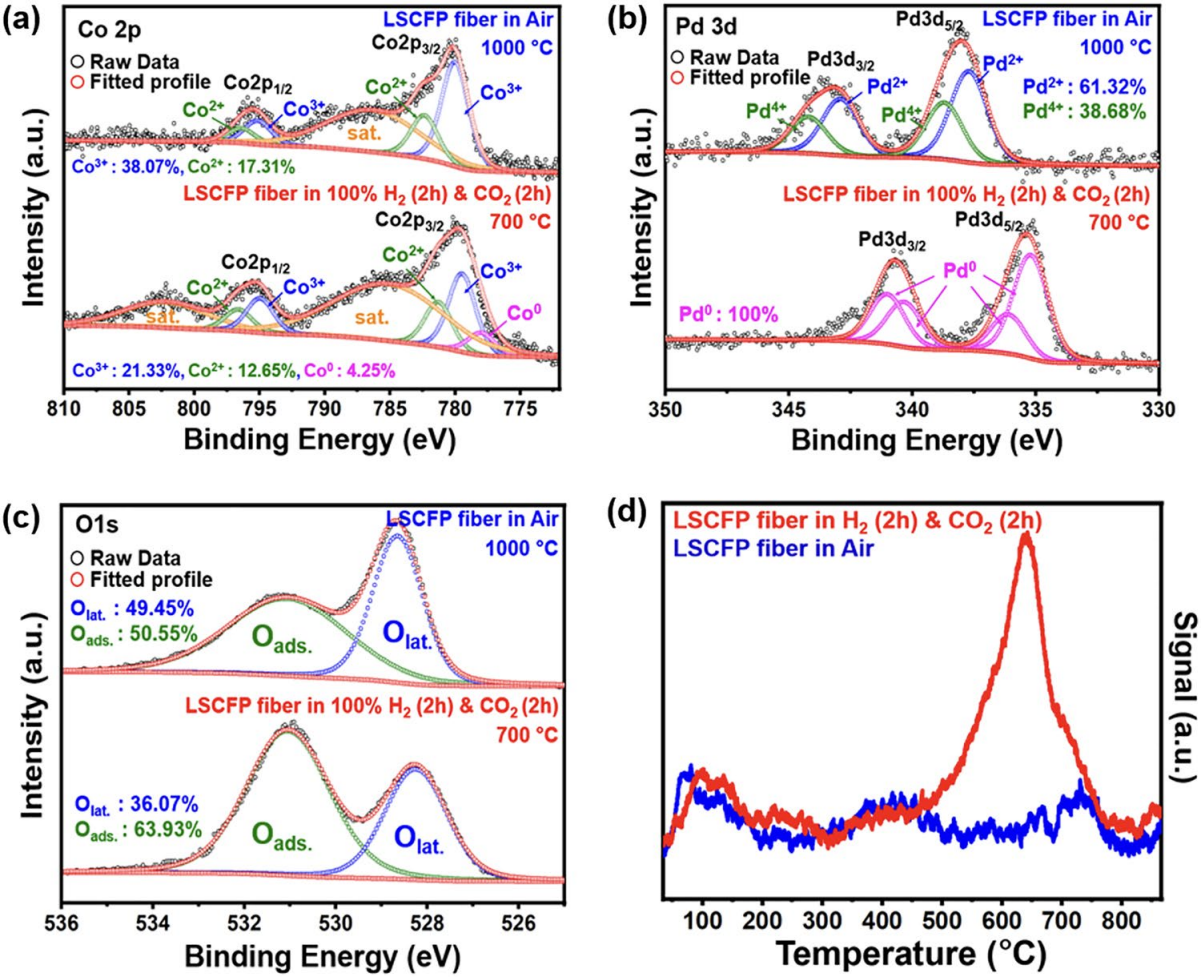
The oxidation state analysis of the LSCFP nanofibers calcined in air (1000 °C for 2 h) and LSCFP nanofibers after consecutive treatment in 100% H_2_ and CO_2_ (700 °C for 2 h each). High-resolution XPS scans of **a** Co 2*p*, **b** Pd 3*d*, and **c** O 1*s* and **d** corresponding CO_2_-TPD profiles

The electrocatalytic activity and selectivity of exsolved catalysts for CO_2_ reduction reaction (CO_2_RR) largely depend on their surface electronic structure [6], which in turn is influenced by many factors, including their oxidation state. We conducted XPS analysis to investigate the oxidation state of the components (La, Sr, Co, Fe, Pd, and O) in the LSCFP nanofibers calcined in air and LSCFP nanofibers consecutively treated in 100% H_2_ and CO_2_. Minimal alterations were observed in the valence state of La 3*d*, Sr 3*d*, and Fe 2*p* spectra before and after the consecutive H_2_-CO_2_ treatment (Fig. S9). Figure 3a shows a high-resolution spectrum of Co 2*p* for both samples. The Co 2*p* spectrum of as-calcined LSCFP nanofibers exhibited two conspicuous bands at 780 and 794.7 eV, assigned to Co 2*p*_3/2_ and Co 2*p*_1/2_, respectively [38, 39]. For LSCFP nanofibers consecutively exposed in 100% H_2_ and CO_2_, the binding energies of 779.4–780 and 795.1 eV corresponded to Co^3+^ [40], while the binding energies of 781.5–782.0 and 796.8 eV were assigned to Co^2+^ [16]. An additional peak was observed at 778.5 eV for the LSCFP nanofibers treated in H_2_ and CO_2_, corresponding to Co^0^, indicating the presence of metallic Co [41]. Similarly, Fig. 3b compares the high-resolution spectrum of Pd 3*d* for both samples. The total Pd content of the LSCFP nanofibers calcined in air comprised Pd^2+^ and Pd^4+^, accounting for 62% and 38%, respectively [42]. After consecutive H_2_ and CO_2_ treatment, however, Pd^4+^ and Pd^2+^ ions changed to metallic Pd with characteristic binding energies located at 334.6, 335.7, 340.0, and 340.8 eV [16, 43]. The O 1*s* spectra of the samples (Fig. 3c) were deconvoluted into two fitting peaks at 528.4 and 531 eV, attributed to two distinct oxygen species: low-energy lattice oxygen (O_L_) and high-energy adsorbed oxygen (O_A_), respectively [44]. The concentration of adsorbed oxygen-containing species associated with oxygen-containing species on the surface of the material for the LSCFP nanofibers consecutively treated in H_2_ and CO_2_ was 63.93%, around 13% higher than of the LSCFP fibers calcined in air, which accounted for 50.55%. These weakly bounded O_A_ ions are known to serve as active sites for CO_2_ adsorption and facilitate electron transfer from the catalyst surface to the CO_2_ molecule, which is a critical step in CO_2_ activation [45, 46].

To investigate the impact of the presence of the catalysts and adsorbed oxygen species on the CO_2_ adsorption/desorption ability of nanofibers, CO_2_*-*TPD profiles of both samples were analyzed (Fig. 3d). CO_2_ adsorption on the electrode surface is generally classified into low-temperature physical adsorption (below 400 °C) and high-temperature chemical absorption (above 400 °C) [47]. Physical adsorption is associated with dipole–dipole coupling between CO_2_ molecule and sample surface via van der Waals force [48], while chemical adsorption is related to the decomposition of bidentate carbonates [49]. Both samples exhibited similar physical desorption peaks at 100 °C. However, the LSCFP nanofibers after consecutively treated in H_2_ and CO_2_ had significantly higher chemical desorption peak intensity and area between 600 and 800 °C than those of LSCFP nanofibers calcined in air, indicating superior CO_2_ adsorption ability. This increase in chemical desorption can be attributed to the larger number of available adsorption sites for CO_2_ molecule incorporation and strong adsorption of the molecule with exsolved nanoparticles on the nanofiber surface, expecting an increased CO_2_RR rate and efficiency [50].

### CO_2_ Reduction Reaction Kinetics

To enhance both electrocatalytic activity and robustness of the nanofiber-based LSCFP fuel electrode for CO_2_-SOEC, we devised a hybrid electrode structure by incorporating crushed LSCFP nanofibers into the original nanofiber matrix (H-LSCFP). Figure S10 schematically illustrates the distinctions in the structure and electrochemical reactions between F-LSCFP and H-LSCFP electrodes. Compared to the excessively porous electrode composed solely of LSCFP nanofibers (F-LSCFP), the addition of crushed nanofibers was expected to improve contact adhesion at the electrode/electrolyte interface and to create additional surface area, which would increase the number of reaction sites available for CO_2_RR. To evaluate the electrochemical activity of the H-LSCFP and F-LSCFP electrodes toward CO_2_RR, we measured the interfacial polarization resistances (*R*_p_) of the electrodes using symmetric cells in 100% CO_2_ of 50 sccm at 700–850 °C and 1.5 V. Prior to CO_2_ measurements, both electrodes were treated with 100% H_2_ of 100 sccm at 700 °C to promote the exsolution of the metallic nanoparticles. It is noteworthy that F-LSCFP and H-LSCFP exhibited consistent CO_2_ desorption peaks, which can be attributed to the shared base material, LSCFP, common to both electrodes (Fig. S11).

**Fig. 4:**
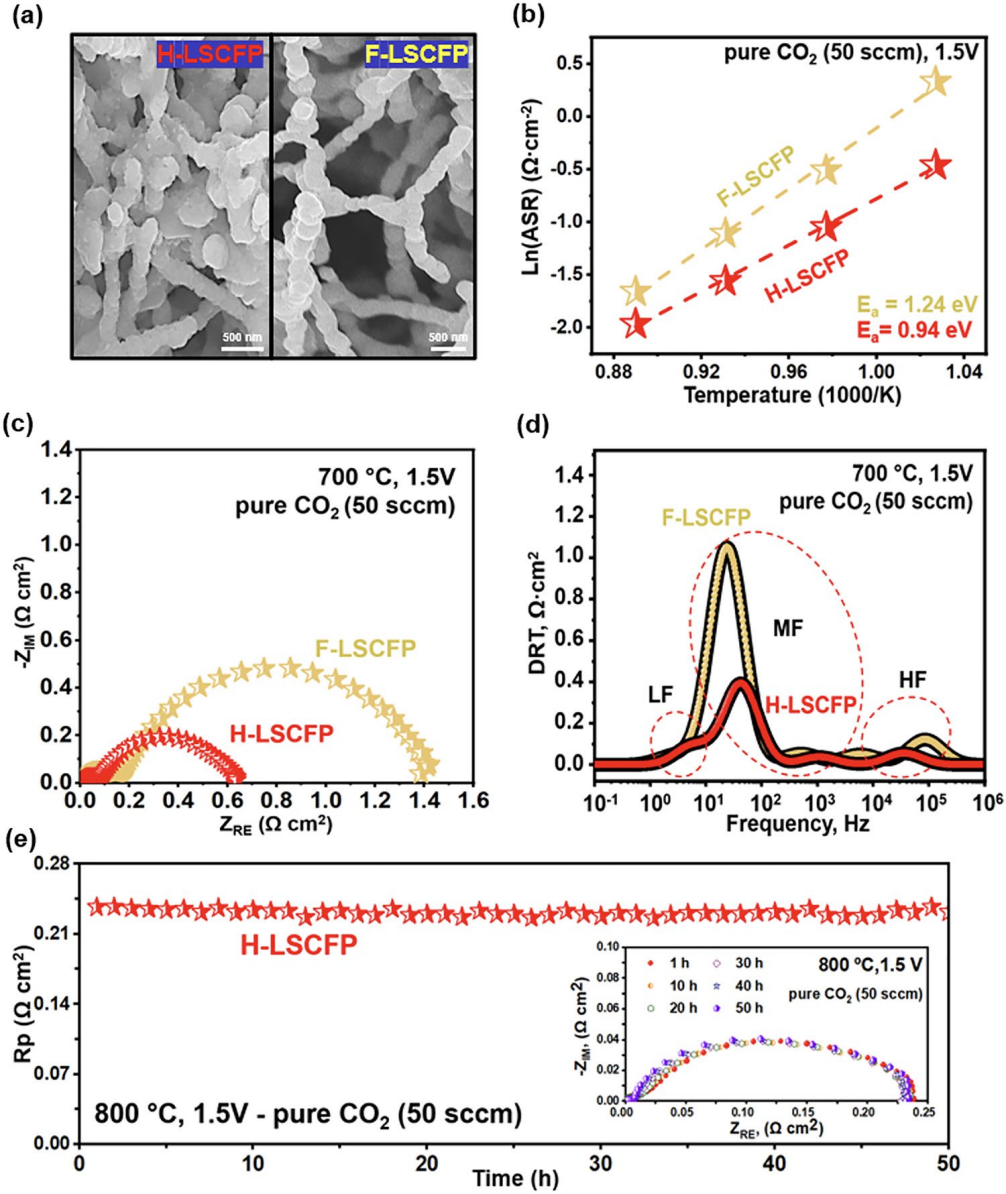
SEM images of the LSGM-supported symmetric cells with **a** H-LSCFP electrode with hybrid and F-LSCFP electrode with 100% nanofiber structures. **b** Temperature dependence of the interfacial polarization resistance (*R*_p_) of both electrodes determined from the impedance data measured between 700 and 850 °C. **c** The impedance spectra of the electrodes in 100% CO_2_ (50 sccm) at 700 °C, 1.5 V and **d** the corresponding DRT functions. **e** Stability test of the LSGM-supported symmetrical cell with H-LSCFP electrode at 800 °C in 100% CO_2_

In Fig. 4a, a magnified SEM image shows the structure of both H-LSCFP and F-LSCFP electrodes. Figure 4b exhibits the temperature-dependent variation in Rp for each electrode during the CO_2_RR reaction within the temperature range of 700–850 °C. The corresponding electrochemical impedance spectroscopy (EIS) and a summary of the values are provided in Fig. S12 and Table S1, respectively. Among all measured temperatures, the H-LSCFP electrode exhibited smaller *R*_p_ with lower activation energy, *E*_a_ (calculated from the slope of the Arrhenius plot) compared to those of F-LSCFP. For instance, at 700 °C (Fig. 4c), the *R*_p_ of H-LSCFP (0.63 Ω cm^2^) was 54% lower than that of F-LSCFP (1.38 Ω cm^2^).

To gain further insight into the kinetics of the complex CO_2_RR, impedance data from both electrodes were deconvoluted into three distinct peaks (Fig. 4d) labeled low frequency (LF), middle frequency (MF), and high frequency (HF) using the DRT method. Each frequency domain corresponds to different electrochemical processes involved in the electrode reaction [51–53], while the integral area represents resistance associated with them [54]. The process contributing to the LF peak (1–10 Hz) represents mass transport process, such as gas diffusion within the electrode [55]. Both electrodes showed minimal mass-transfer resistances, most likely due to porous electrode structure that facilitates mass transfer. The process in the MF range (10 Hz–1 kHz) is likely associated with CO_2_ adsorption/desorption, dissociation, surface transport, as well as coupling of these processes [55, 56]. These processes dominated the total polarization resistance in both electrodes. Nonetheless, the integral area of the H-LSCFP was significantly smaller than that of the F-LSCFP, indicating improved kinetics of the H-LSCFP toward the given process. This improvement is probably attributed to the high specific surface area achieved by combination of crushed LSCFP nanofibers and original nanofibers. The process contributing to the peak in the HF region (10–100 kHz) is associated with the oxygen ion transfer through the electrode/electrolyte interface and incorporation into the electrolyte [55]. Although these processes made minimal contributions to the total resistance of both electrodes, the H-LSCFP exhibited relatively smaller resistance to these processes.

Figure 4e shows a short-term stability test of the symmetrical cell with the H-LSCFP electrode at 800 °C in CO_2_ of 50 sccm under a constant applied potential of 1.5 V, with the inset displaying EIS plot at 10 h intervals. The resultant ASR value remained close to the initial value of 0.24 Ω cm^2^ over 50 h of operation, demonstrating the high durability of the H-LSCFP against the CO_2_ environment.

### CO_2_ Electrolysis Performance

**Fig. 5:**
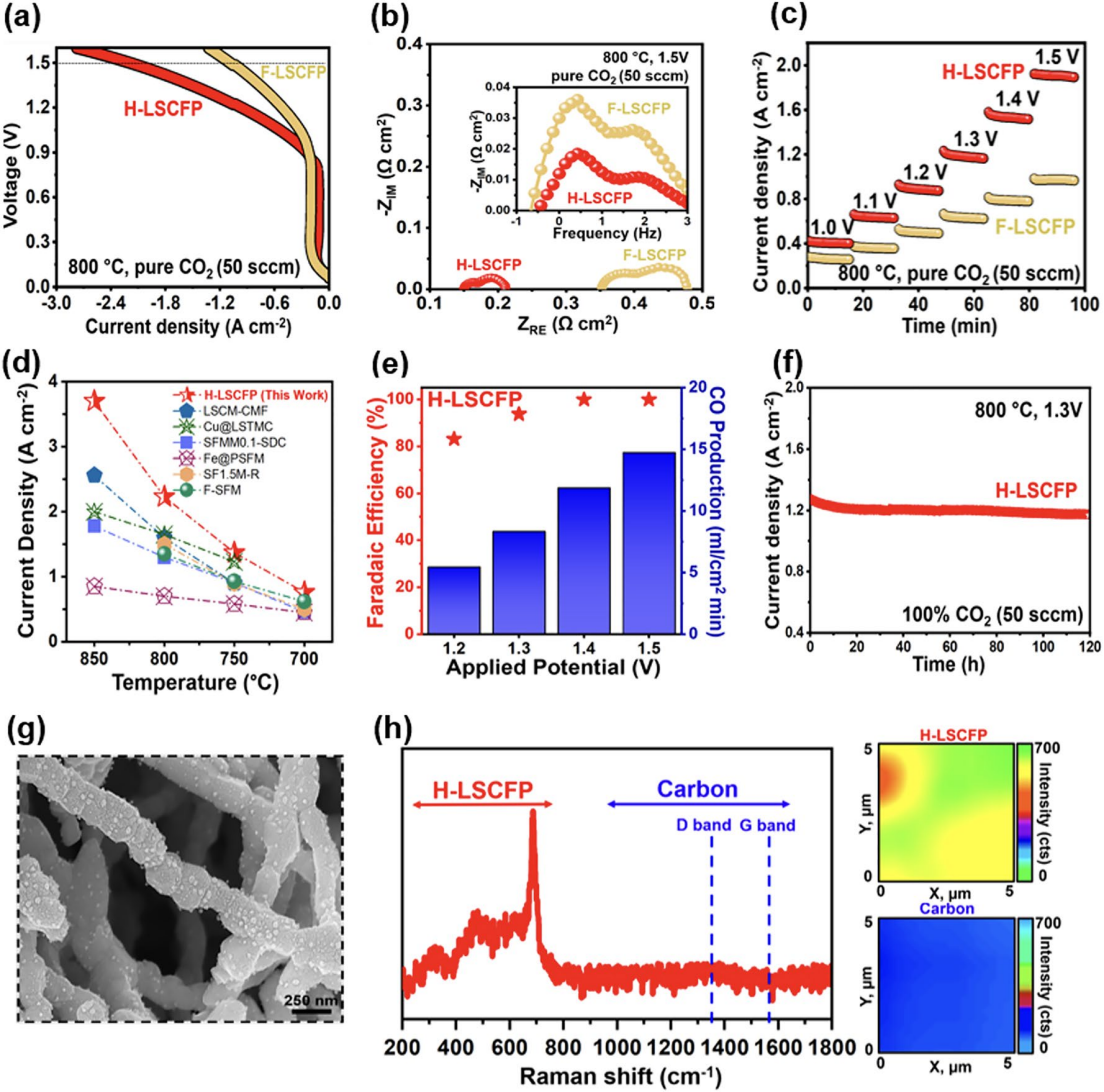
**a** *I–V* curves of the LSGM-supported singles cells with H-LSCFP and F-LSCFP electrodes at 800 °C under 100% CO_2_ of 50 sccm and **b** the corresponding Nyquist plots (inset: Bode plots). **c** Potentiostatic tests of the corresponding cells. **d** Electrochemical performance comparison in current density obtained at 1.5 V with the reported high-performance fuel electrodes. **e** Faradaic efficiency and CO production rate during the CO_2_RR of the SOEC with the H-LSCFP fuel electrode within an applied potential range of 1.2–1.5 V, in 100% CO_2_ of 50 sccm. **f** Stability test of the LSGM-supported single cell with the H-LSCFP electrode in 100% CO_2_ at 800 °C and 1.3 V and **g** magnified microstructure SEM image of the corresponding cell. **h** Raman spectra collected from H-LSCFP electrode after 100 h long-term analysis

The performance of H-LSCFP and F-LSCFP fuel electrodes in CO_2_ electrolysis was compared using LSGM electrolyte-supported single cells with identical LSCF-GDC oxygen electrodes. Prior to CO_2_ measurements, both fuel electrodes were treated with 100% H_2_ of 100 sccm at 700 °C for 2 h to promote the exsolution of metallic nanoparticles. Figure 5a shows the current density of the SOEC with the H-LSCFP and F-LSCFP fuel electrodes measured in 100% CO_2_ of 50 sccm at 800 °C with an applied potential scanned from open-circuit voltage (*V*_oc_) to 1.6 V. The H-LSCFP cell exhibited a maximum current density of 2.2 A cm^−2^ at 1.5 V, approximately twice that of the F-LSCFP cell (1.09 A cm^−2^). To demonstrate the broader applicability of the H-LSCFP electrode, solid oxide cells (SOCs) with H-LSCFP and F-LSCFP fuel electrodes were tested in the fuel cell (FC) mode within the temperature range of 650–800 °C, where the H-LSCFP outperformed the F-LSCFP cell across all tested temperatures (Fig. S13). The MPD values are summarized in Table S2. This difference in performance can be attributed to the microstructure differences between H-LSCFP and F-LSCFP fuel electrodes, as the rest of the cell configuration was identical. Figure 5b displays the corresponding Nyquist and Bode plots of EIS data of both cells at 1.5 V. In the Nyquist plots, the area specific ohmic (ASR_ohm_) and electrode (ASR_elec_) resistances were evaluated from the high-frequency intercept and the difference between the high and low frequency intercepts on the real axis, respectively [33, 57]. The H-LSCFP cell showed roughly 52% lower ASR_elec_ (0.061 Ω cm^2^) than that of F-LSCFP (0.127 Ω cm^2^). Moreover, the ASR_ohm_ of the H-LSCFP cell (0.149 Ω cm^2^) was approximately 2.4 times lower than that of the F-LSCFP (0.352 Ω cm^2^). Considering the identical thickness of the LSGM electrolyte, the difference in the ASR_ohm_ can be attributed to disparities in the contact adhesion between the H-LSCFP and F-LSCFP fuel electrodes at the LSGM electrolyte interface, as confirmed by the cross-sectional image of both electrodes (Fig. S14). The impedance responses of both cells were further compared using Bode plots shown in the inset of Fig. 5b. Both cells exhibited a dominant large semicircular arc in the low-frequency region related to the CO_2_ surface adsorption/desorption processes and smaller semicircular arc in the high-frequency region associated with the oxygen ion transfer process at the electrode/electrolyte interface [1]. The integral area of the SOEC with the H-LSCFP fuel electrode was significantly smaller than that of the SOEC with the F-LSCFP, indicating improved CO_2_RR kinetics and better contact adhesion of the H-LSCFP electrode on the electrolyte surface. Figure 5c shows the current density values obtained from a potentiostatic stability test of both SOECs in 100% CO_2_ of 50 sccm at 800 °C and a voltage range of 1.0–1.5 V. The H-LSCFP cell yielded significantly higher current density values than the F-LSCFP cell at all tested potentials. Specifically, the current density of the H-LSCFP cell (1.18 A cm^−2^) at 1.2 V was ~ 87% higher than that of the F-LSCFP cell (0.63 A cm^−2^), with the remaining results summarized in Table S3. Additional short-term potentiostatic stability test was conducted at 750 °C in 100% CO_2_ (50 sccm), within a potential range of 1.0–1.4 V, to verify the effect of the exsolution treatment on CO_2_ electrolysis performance of the H-LSCFP cell. Following exsolution, the H-LSCFP cell consistently demonstrated significantly higher current density values across all tested potentials (Fig. S15) when compared to the H-LSCFP cell without exsolution, as summarized in Table S4.

Figure S16 exhibits the temperature-dependent performance of the SOEC with the H-LSCFP and F-LSCFP fuel electrodes over a temperature range of 700–850 °C. The H-LSCFP cell achieved maximum current density values of 3.7, 2.2, 1.36, and 0.76 A cm^−2^ at 850, 800, 750, and 700 °C, respectively, under applied potential of 1.5 V. These results exhibited significantly higher current densities compared to those of the F-LSCFP cell (1.7, 1.09, 0.66, and 0.42 A cm^−2^), respectively, following the same temperature order. It is worth noting that, at all temperatures, the H-LSCFP cell exhibited smaller ASR_ohm_ and ASR_elec_ values compared to the F-LSCFP cell (Fig. S17). Figure 5d and Table 2 compare these values with various perovskite-based fuel electrodes reported in the literature as a function of temperature [1, 3, 4, 6, 18, 46, 58, 59], where the H-LSCFP cell significantly outperformed state-of-the-art ceramic fuel electrodes for CO_2_-SOEC at all tested temperatures. To validate the high performance of the H-LSCFP cell, we conducted multiple runs of the identical experiment using different H-LSCFP cells, thus confirming the robustness and reproducibility of the reported results (Fig. S18).

**Table 2:** A comparison of current density values obtained during CO_2_ electrolysis at 800 °C and 1.5 V between various perovskite-based and Ni-based fuel electrodes

Cell configuration				Maximum current density at 1.5 V (A cm^2^)				Refs.
Air electrode	Electrolyte	Buffer layer	**Fuel electrode**	850 °C	800 °C	750 °C	700 °C	
LSCF-GDC	LSGM^b^	LDC^e^	**H-LSCFP**^c^	3.7	2.2	1.37	0.76	This work
SSC^l^	LSGM^b^	LDC^e^	**LSCM**^f^**-CMF**^h^	2.56	1.6	1.0	–	[1]
LSCF^a^	LSGM^d^	LDC	**Cu@LSTMC**^k^	2.0	1.65	1.2	–	[47]
LSCF^a^-SDC^i^	LSGM^d^	–	**SFMM0.1**^r^**-SDC**^i^	1.78	1.3	0.91	0.46	[48]
LSCF^a^-GDC^j^	LSGM^b^	LDC^g^	**Fe@PSFM**^m^	0.85	0.7	0.58	0.45	[4]
LSCF^a^-SDC^i^	LSGM^b^	LDC^e^	**SF1.5M**^o^**-R**	–	1.5	0.9	0.5	[3]
LSCF-SDC	LSGM	LDC	**F-SFM**^n^	–	1.36	0.93	0.62	[6]
BLC^s^	LSGM^d^	–	**Ni–Fe-LSFM**^p^	–	1.5	–	0.6	[16]
LSCF^a^-GDC	YSZ	–	**LSFNF0.1**^**q**^**-GDC**	1.25	0.9	0.6	–	[34]

^a^LSCF: La_0.6_Sr_0.4_Co_0.2_Fe_0.8_O_3−*δ*_; ^b^LSGM: La_0.8_Sr_0.2_Ga_0.8_Mg_0.2_O_3−*δ*_; LSCFP: ^c^La_0.6_Sr_0.4_Co_0.15_Fe_0.8_Pd_0.05_O_3−*δ*_; ^d^LSGM: La_0.9_Sr_0.1_Ga_0.8_Mg_0.2_O_3−*δ*_; ^e^LDC: Ce_0.6_La_0.4_O_2−*δ*_; ^f^LSCM: La_0.75_Sr_0.25_Cr_0.5_Mn_0.5_O_3−*δ*_; ^g^LDC: Ce_0.5_La_0.5_O_0.175_; ^h^CMF: Ce(Mn,Fe)O_2_; ^i^SDC: Ce_0.8_Sm_0.2_O_1.9_; ^j^GDC: Gd_0.1_C_0.9_O_1.95_; ^k^LSTMC: (La_0.2_Sr_0.8_)_0.9_Ti_0.5_Mn_0.4_Cu_0.1_O_3−*δ*_; ^l^SSC: Sm_0.5_Sr_0.5_CoO_3_; ^m^PSFM: Pr_0.4_Sr_0.6_Fe_0.875_Mo_0.125_O_3–*δ*_; ^n^F-SFM: Sr_2_Fe_1.5_Mo_0.5_O_6−*δ*_F_0.1_; ^o^SF1.5 M: Sr_2_Fe_1+*x*_Mo_1−*x*_O_6−*δ*_; ^p^Ni–Fe–LSFM: Ni–Fe–La_0.6_Sr_0.4_Fe_0.8_Mn_0.2_O_3−*δ*_; ^q^LSFNF0.1: La_0.6_Sr_0.4_Fe_0.8_Ni_0.2_O_2.9−*δ*_F_0.1_; ^r^SFMMx: Sr_2_Fe_1.5−*x*_Mn_*x*_Mo_0.5_O_6−*δ*_; ^s^BLC: Ba_0.6_La_0.4_Co_1.0_O_3−*δ*_

The CO_2_ conversion, including CO production rate and Faradaic efficiency, is essential for assessing the performance and practical viability of SOECs. Figure 5e shows the off-gas analysis results from the LSGM-supported SOEC with the H-LSCFP electrode at 800 °C, within an applied potential range of 1.2–1.5 V, in 100% CO_2_ of 50 sccm. The high average Faradaic efficiency, reaching approximately 94%, and the significant CO production rate strongly indicate that CO was the primary product generated during the CO_2_RR, demonstrating the suitability of the H-LSCFP cell for direct CO_2_ electrolysis application.

Figure 5f shows the long-term operation of the H-LSCFP cell in 100% CO_2_ of 50 sccm at 800 °C and under a constant applied potential of 1.3 V. After an initial stabilization period, the H-LSCFP cell maintained stable performance over 100 h, demonstrating the high durability in the CO_2_ environment. In addition, stability test of the SOEC with H-LSCFP-based electrode in EC mode is presented in Fig. S19, showing stable current density output over 240 h. Figure S20 shows cross-sectional SEM image of the H-LSCFP cell after long-term stability test, demonstrating good bonding between all components. Moreover, Fig. 5g displays a magnified SEM image of the H-LSCFP electrode’s surface after the 100-h long-term test, covered with in situ exsolved nanoparticles and the notable absence of carbon formation. TEM–EDX and point analyses revealed that the exsolved nanoparticles predominantly consisted of Co, with occasional instances of Pd nanoparticle, while the stoichiometry of the nanofiber body remained close to the initial state (Fig. S21). Moreover, the XRD analysis of the H-LSCFP cell after the long-term stability test further revealed the presence of a perovskite phase, along with additional phases of Co and Pd (Fig. S22). Furthermore, Fig. 5h shows Raman spectra with Raman mappings of the H-LSCFP and carbon formation following long-term operation. The reduced H-LSCFP perovskite electrode exhibited spectra in the range of 100–750 cm^−1^ without any observed carbon coking at 1359 and 1581 cm^−1^ [1, 7], demonstrating the H-LSCFP electrode’s high durability against CO_2_. Furthermore, Fig. S23 shows the XPS analysis conducted to investigate the chemical stability and any changes in the components (La, Sr, Co, Fe, Pd and O) of the H-LSCFP electrode before and after the 100-h stability. Comparing the peak positions of the spin orbitals for La 3*d*, Sr 3*d*, and Fe 2*p*, negligible differences were observed. In contrast, the Co 2*p* and Pd 3*d* spectra after the test exhibited peaks indicating the presence of metallic Co and Pd nanoparticles. Moreover, the concentration of the oxygen-containing species adsorbed on the surface (O_A_) was relatively higher than that of the lattice oxygen (O_L_), consistent with the observations from both XRD (Fig. 2) and XPS (Fig. 3) conducted before the test.

### Digital Twinning

**Fig. 6:**
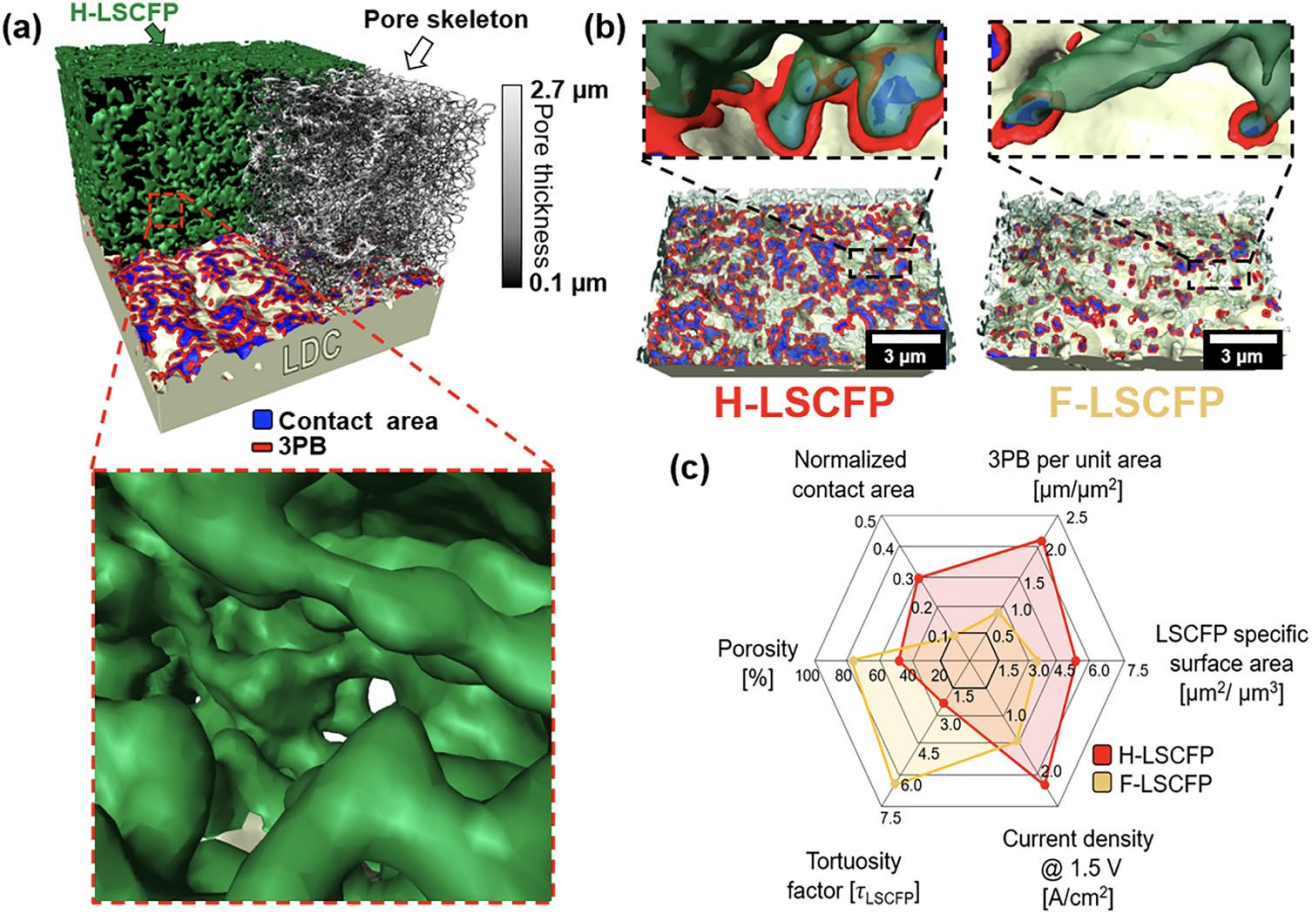
**a** 3D reconstructed architecture and magnified 3D microstructure image of the H-LSCFP electrode composed of LSCFP nanofibers and LSCFP crushed fibers. **b** Detailed view comparing TPB between the H-LSCFP and F-LSCFP at the electrode/buffer layer interface. **c** Quantitative comparison of the microstructural and electrochemical properties of the H-LSCFP and H-LSCSFP electrodes using a radar plot

The correlation between microstructural characteristics of the fuel electrode and CO_2_ electrolysis performance was elucidated through the digital twinning of both nanofiber-based electrode using a state-of-the-art 3D reconstruction technique with a FIB-SEM dual beam system (Fig. S24). Figure 6a depicts a 3D reconstructed image of the H-LSCFP electrode, showcasing the contact area between the electrode and LDC buffer layer, triple phase boundary (TPB), and pore skeleton. Within the uniformly connected porous network of the electrode, the skeletonized pore demonstrates a distributed pore thickness in the range from 0.1 to 2.7 µm. Table S5 summarizes the quantified 3D microstructural features, including volume fraction, tortuosity factor, phase connectivity, surface area, and TPB values analyzed from the total volumes of H-LSCFP (3398 µm^3^) and F-LSCFP (3,639 µm^3^) electrode samples displayed in Fig. S25. A magnified view in Fig. 6a reveals the hybrid structure of the H-LSCFP electrode, wherein the incorporation of crushed nanofibers into the original nanofiber matrix significantly enhanced both the tortuosity factor (2.4) and volume fraction (48.7%) compared to the excessively porous F-LSCFP electrode, composed solely of nanofibers (tortuosity factor of 6.6 and volume fraction of 21.6%). This enhancement could facilitate O^−2^ ion transport within the H-LSCFP electrode.

Figure 6b visually compares the contact area and TPB density of both fuel electrodes at the electrode/LDC buffer layer interface. The contact area plays a vital role in the incorporation of O^2−^ ions into the oxygen conducting phase, while the TPB is crucial for electrochemical reduction of CO_2_ molecules. The normalized contact area (0.29) and TPB per unit area (2.08 µm µm^−2^) of the H-LSCFP were 190% and 147% higher than the contact area (0.1) and TPB (0.84 µm µm^−2^) of the F-LSCFP, respectively. Figure 6c presents a radar plot based on quantified microstructural properties and electrochemical performances of SOECs with both electrodes. Although the F-LSCFP electrode exhibited higher porosity for facile gas diffusion, the well-tailored microstructure of the H-LSCFP electrode, characterized by lower tortuosity factor, better contract adhesion, higher surface area, and higher TPB length, resulted in facile O^2−^ migration, higher catalytic activity, and robustness for CO_2_RR. Additionally, to assess the microstructural stability of the H-LSCFP electrodes, we also conducted analyses on samples collected after 100 h long-term testing. Figure S26 illustrates the reconstructed volume, and the corresponding quantitative data can be found in Table S6. Minor differences in quantified values, all within a margin of less than 10%, were observed between before and after the 100 h test. These slight variations can be attributed to heterogeneity of the electrode. Importantly, the overall microstructural characteristics remained largely unchanged even after prolonged exposure to high-temperature CO_2_ electrolysis.

## Conclusion

In this study, we developed a novel hybrid structured nanofiber electrode for CO_2_-SOEC application by integrating crushed LSCFP nanofibers into the high aspect ratio nanofiber framework synthesized using the electrospinning technique. Comprehensive physicochemical analyses revealed that after consecutive treatment in 100% H_2_ and CO_2_ at 700 °C, the LSCFP nanofibers underwent in situ exsolution, resulting in the formation of a perovskite structure and the decoration of metallic Co (majority) and Pd (minority) nanoparticles on the surface, while exhibiting a high concentration of surface oxygen species, enhancing the CO_2_ adsorption ability. Furthermore, the SOEC with the H-LSCFP electrode demonstrated a remarkable twofold increase in current density (2.2 A cm^−2^) compared to the F-LSCFP cell (1.09 A cm^−2^) in 100% CO_2_ at 800 °C and 1.5 V, indicating significantly improved CO_2_ surface adsorption/desorption kinetics of the H-LSCFP electrode. To the best of our knowledge, this result ranks among the top-tier performances reported to date for perovskite fuel electrodes in a similar SOEC configuration. Moreover, the quantitative 3D reconstruction analysis to create a digital twin of the nanofiber structured electrodes revealed that the H-LSCFP electrode exhibited a 190% increase in the normalized contact area and a 147% increase in the TPB per unit area compared to the F-LSCFP electrode. These enhancements were found to be highly correlated with the improved electrochemical performances. Overall, our results demonstrate the viability of achieving a highly catalytically active and durable nanofiber-based fuel electrode with a hybrid structure, setting the stage for further advancements and broader utilization of nanofibers in CO_2_-SOEC applications.

## Supplementary Information

Below is the link to the electronic supplementary material.

Supplementary file1 (PDF 2906 kb)
