# Supplementary material for: Novel Perovskite Oxide Hybrid Nanofibers Embedded with Nanocatalysts for Highly Efficient and Durable Electrodes in Direct CO2 Electrolysis
Source: Nanomicro Lett. 2024 Jan 22;16:93. doi: 10.1007/s40820-023-01298-w (PMC10803691; doi:10.1007/s40820-023-01298-w)
Supplement: Supplementary file 1 — Supplementary file1 (PDF 2906 kb) [file 40820_2023_1298_MOESM1_ESM.pdf]

Supporting Information for

Novel Perovskite Oxide Hybrid Nanofibers Embedded with Nanocatalysts for Highly Efficient and Durable Electrodes in Direct CO_2 Electrolysis

Akhmadjonov Akromjon[1, †], Kyung Taek Bae[1, †], and Kang Taek Lee[1, 2, *]

[1] Department of Mechanical Engineering, KAIST, Daejeon 34141, Republic of Korea

[2] KAIST Graduate School of Green Growth & Sustainability, Daejeon 34141, Republic of Korea

† Akhmadjonov Akromjon and Kyung Taek Bae contributed equally to this work.

* Corresponding author. E-mail: leekt@kaist.ac.kr (Kang Taek Lee)

Supplementary Figures and Tables

Table S1 Electrode ASR values of H-LSCFP and F-LSCFP measured in 100% CO_2 of 50 sccm at 1.5 V within the temperature range of 700~850 °C

		850 °C	800 °C	750 °C	700 °C
ASR (Ω cm^2) in 100% CO_2	H-LSCFP	0.15	0.21	0.35	0.64
	F-LSCFP	0.19	0.33	0.6	1.38

Table S2 Comparison of the MPD values between H-LSCFP and F-LSCFP cells within the temperature range of 650~800 °C

		800 ˚C	750 ˚C	700 ˚C	650 ˚C
MPD (W cm^{-2})	H-LSCFP	1.95	1.44	1.00	0.66
	F-LSCFP	1.27	0.93	0.63	0.43

Table S3 The current density values of the H-LSCFP and F-LSCFP cells obtained during the short-term CO_2 electrolysis test in CO_2 of 50 sccm at 800 °C and a voltage range of 1.0-1.5 V

Fuel electrodes	1.0 V	1.1 V	1.2 V	1.3 V	1.4 V	1.5 V
H-LSCFP	0.4 A cm^2	0.64 A cm^2	0.88 A cm^2	1.18 A cm^2	1.53 A cm^2	1.91 A cm^2
F-LSCFP	0.26 A cm^2	0.36 A cm^2	0.5 A cm^2	0.63 A cm^2	0.78 A cm^2	0.97 A cm^2

Table S4 The current density values of the H-LSCFP cell before and after exsolution obtained during the short-term CO_2 electrolysis test in 100% CO_2 of 50 sccm at 750 °C, with a voltage range of 1.0-1.4V

100% CO_2 at 750 °C		1.0V	1.1V	1.2V	1.3V	1.4V
Current Density(A cm^{-2})	Exsolved H-LSCFP	0.32	0.47	0.65	0.85	1.09
	Pristine H-LSCFP	0.24	0.35	0.47	0.61	0.74

Table S5 Summary of the microstructural parameters of the H-LSCFP and F-LSCFP quantified via 3D reconstruction technique

		H-LSCFP	F-LSCFP
Volume fraction (%)	LSCFP	48.7	21.6
	Pore	51.3	78.4
Tortuosity factor (τ)	LSCFP	2.4	6.6
	Pore	2.1	1.3
Connectivity (%)	LSCFP	99.9	99.7
Two phase area: V ($\mu m^2/\mu m^3$)	LSCFP-Pore	5.4	3.3
Normalized contact area		0.29	0.10
TPB per unit area ($\mu m/\mu m^2$)		2.08	0.84

Table S6 Summary of microstructural parameters of the H-LSCFP fuel electrode after the stability test quantified via 3D reconstruction technique

		H-LSCFP after test
Volume fraction (%)	LSCFP	44.53
	Pore	55.47
Tortuosity factor (τ)	LSCFP	2.53
	Pore	1.98
Connectivity (%)	LSCFP	99.99
Two phase area: V ($\mu m^2/\mu m^3$)	LSCFP-Pore	5.27
Normalized contact area		0.27
TPB per unit area ($\mu m/\mu m^2$)		1.99

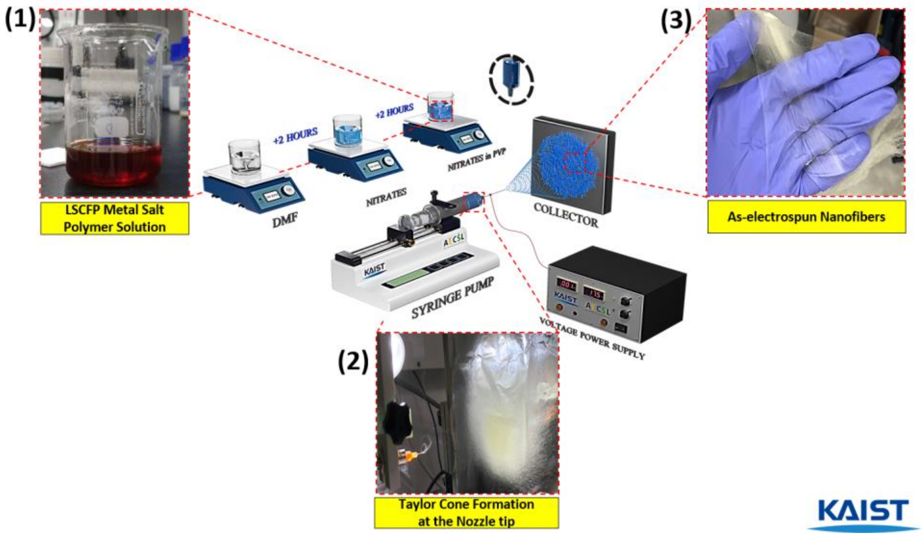

Fig. S1 A step-by-step description of the electrospinning process used to synthesize LSCFP nanofibers

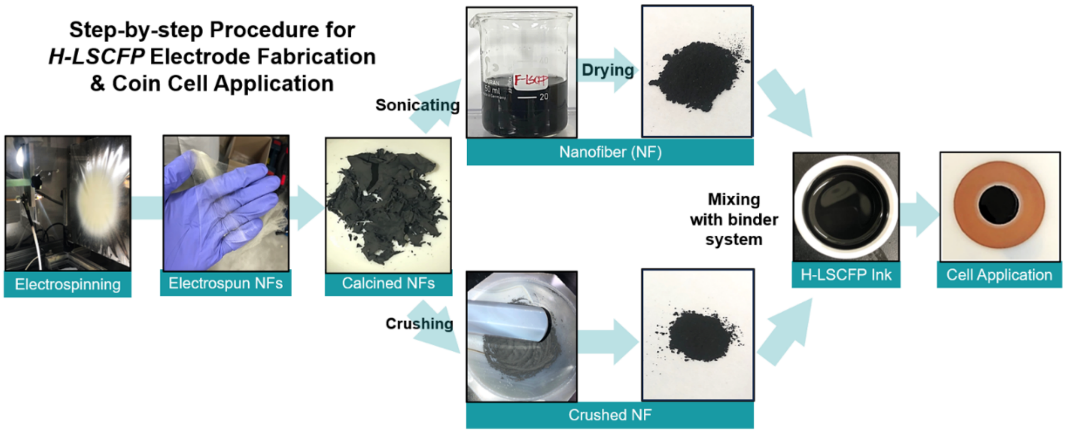

Fig. S2 A step-by-step description of the H-LSCFP electrode fabrication procedure

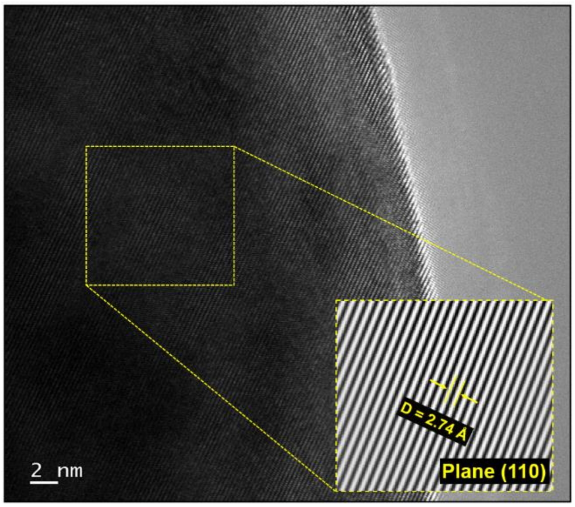

Fig. S3 High-resolution TEM (HR-TEM) image of pristine LSCFP nanofiber with the inset showing a lattice distance of (110) plane

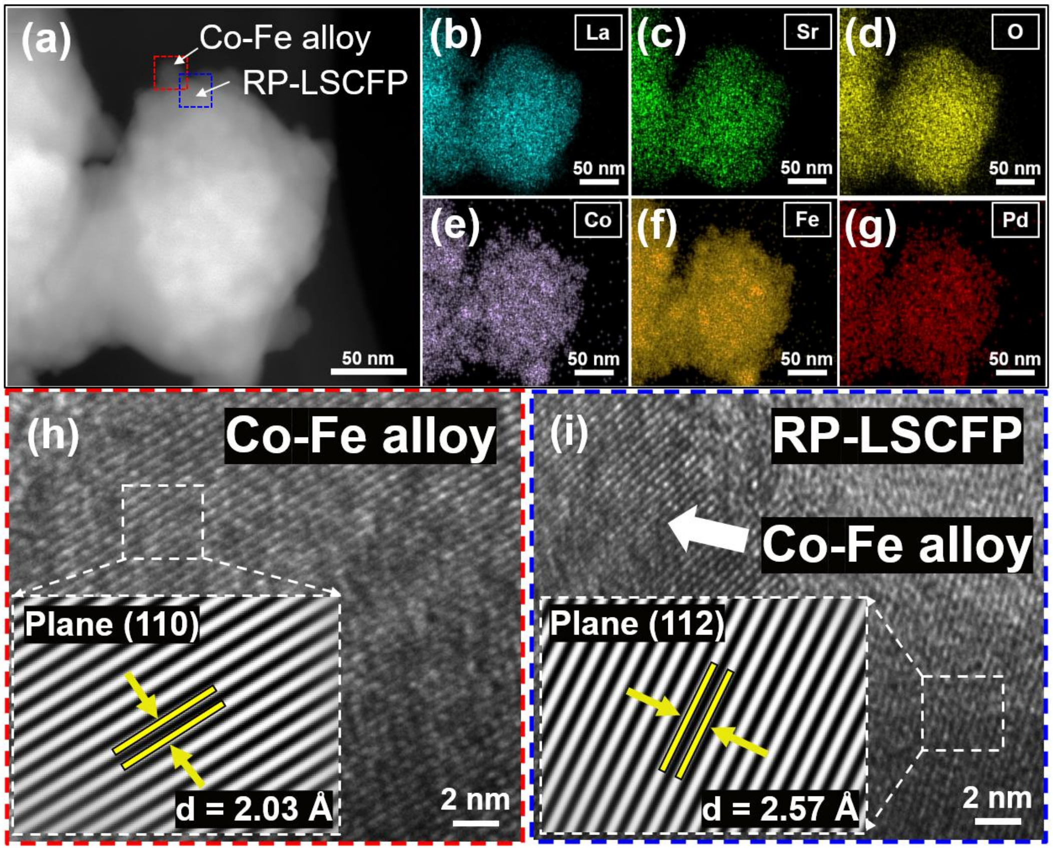

Fig. S4 a STEM image of the LSCFP nanofibers treated in 100% H_2 at 700 °C for 2h and **b-g** corresponding elemental mappings of La (cyan), Sr (green), Co (purple), Fe (dark orange), Pd (red), and O (yellow). Magnified high-resolution TEM images of **h** Co-Fe nanoparticles and **i** RP-LSCFP matrix acquired from the red and blue rectangular region in **a**, respectively. Insets are the lattice fringe patterns

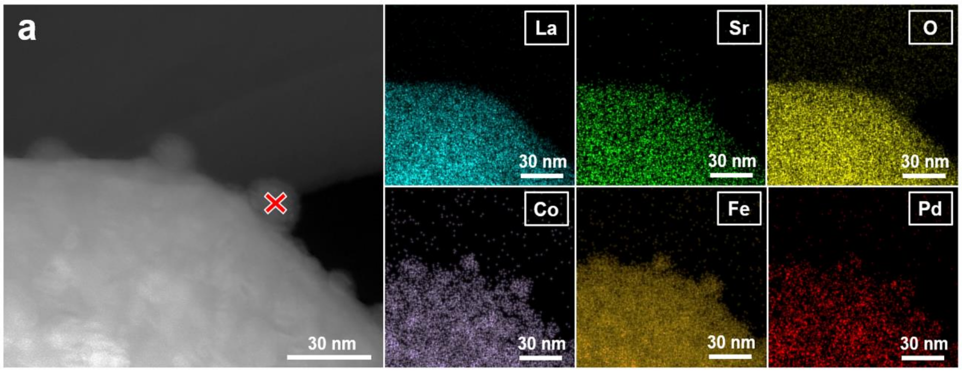

b

Elements	La	Sr	Co	Fe	Pd	O
at %	0.7	0.34	28.56	46.5	11.22	12.6

Fig. S5 a STEM image and corresponding elemental mappings of the LSCFP nanofibers heat treated in 100% H_2 of 100 sccm at 700 °C for 2 h and **b** point-EDX analysis of a nanoparticle exsolved particle on the surface

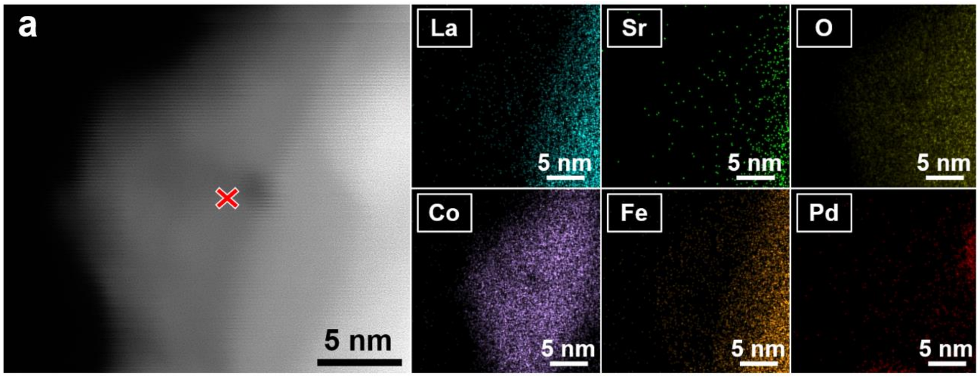

b

Elements	La	Sr	Co	Fe	Pd	O
at %	0.5	0.18	54.3	1.64	0	43.3

Fig. S6 a STEM image and corresponding elemental mappings of the LSCFP nanofibers consecutively treated in 100% H_2 of 100 sccm and 100% CO_2 of 100 sccm at 700 °C for 2 h each and **b** point-EDX analysis of a nanoparticle exsolved particle on the surface

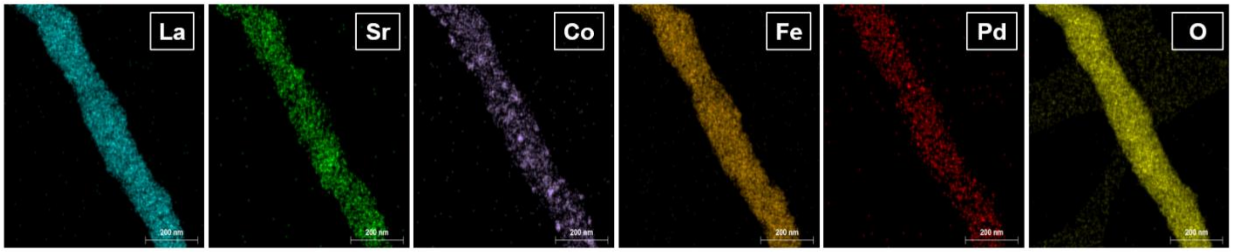

Fig. S7 TEM elemental mapping images of the LSCFP nanofiber after consecutive treatment in 100% H_2 (2 h) and 100% CO_2 (0.5 h)

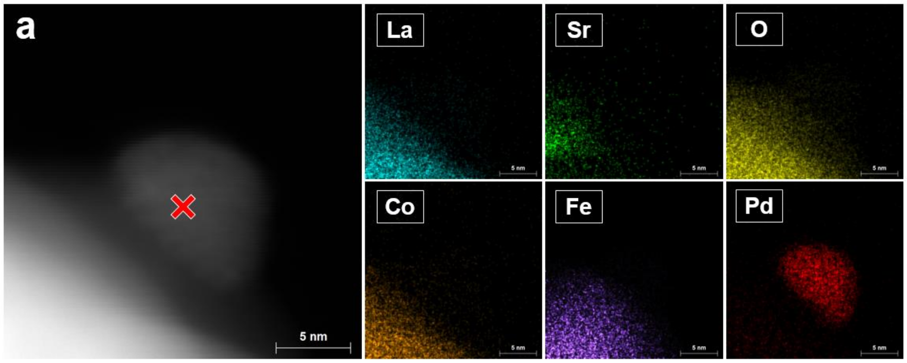

b

Elements	La	Sr	Co	Fe	Pd	O
at %	0.52	0.89	14.77	1.74	71.86	10.21

Fig. S8 a STEM image and corresponding elemental mappings of the LSCFP nanofibers consecutively treated in 100% H_2 of 100 sccm and 100% CO_2 of 100 sccm at 700 °C for 2 h each and **b** point-EDX analysis of a nanoparticle exsolved particle on the surface

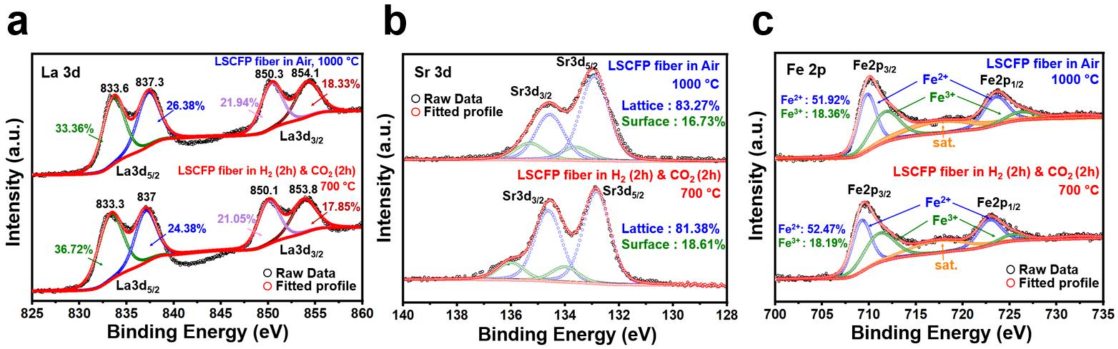

Fig. S9 The oxidation state analysis of the LSCFP nanofibers calcined in air (1000 °C for 2h) and LSCFP nanofibers after consecutive treatment in 100% H_2 and CO_2 (700 °C for 2h each). High resolution XPS scans of **a** La 3d, **b** Sr 3d, and **c** Fe 2p

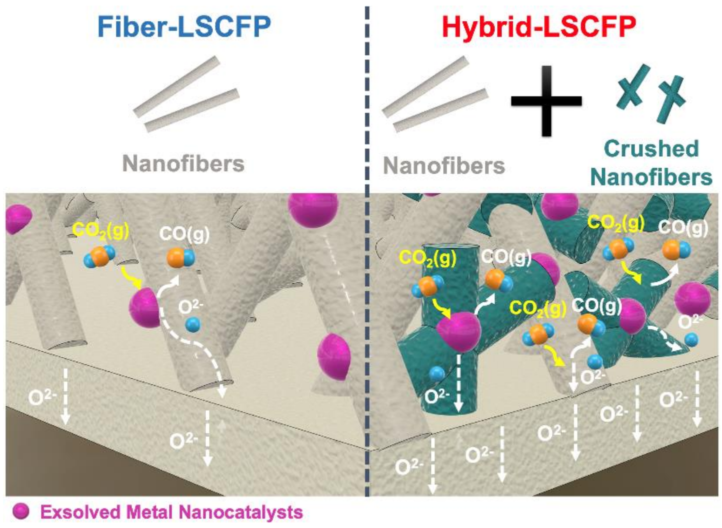

Fig. S10 Schematic diagram illustrating the differences in the structure and electrochemical reactions between F-LSCFP and H-LSCFP electrodes

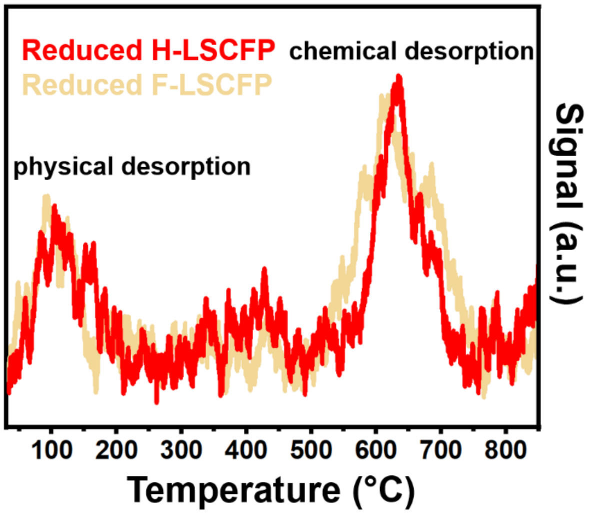

Fig. S11 CO_2-TPD profiles of the F-LSCFP and H-LSCFP electrodes after consecutive treatment in 100% H_2 and CO_2 (700 for 2h each)

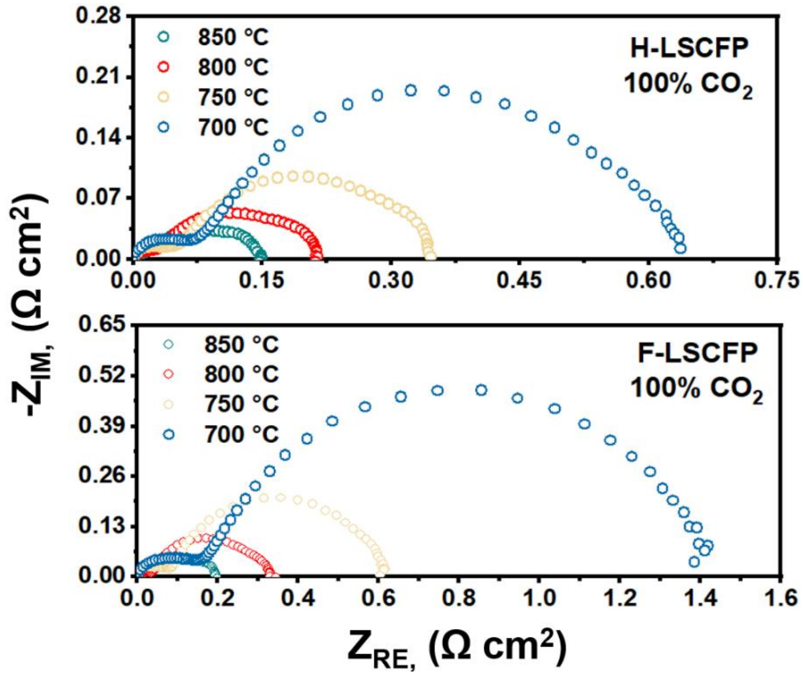

Fig. S12 EIS of the symmetrical cells with the H-LSCFP and F-LSCFP electrodes in 100% CO_2 of 50 sccm at 1.5 V within the temperature range of 700~850 °C

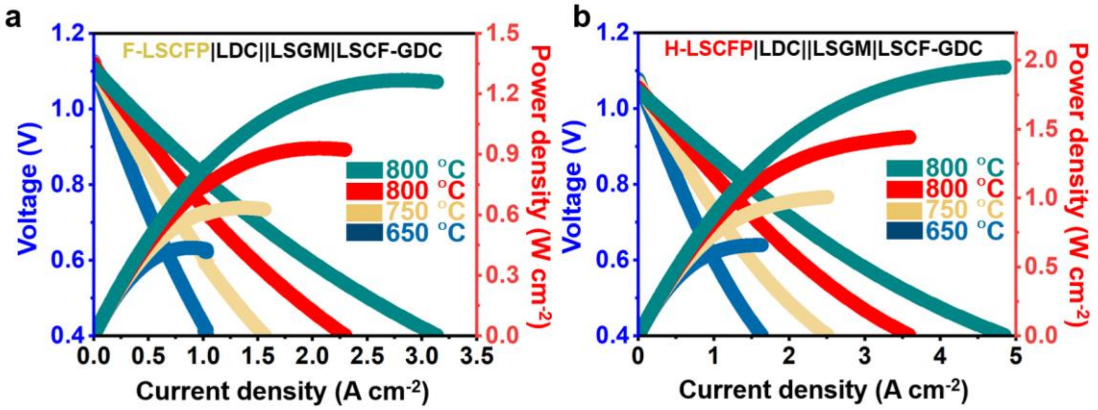

Fig. S13 *I-V-P* curves of the single cells with the (**a**) F-LSCFP, and (**b**) H-LSCFP fuel electrodes at 650-800 °C in FC mode

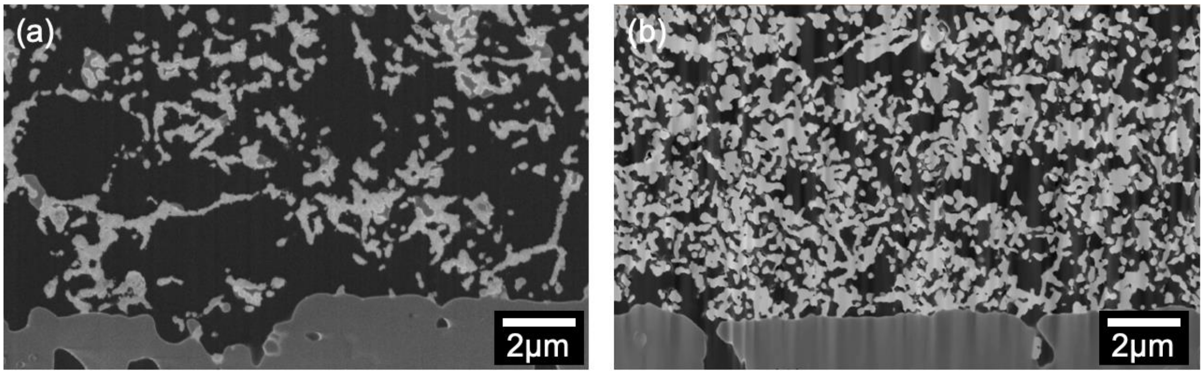

Fig. S14 Cross sectional images of (**a**) F-LSCFP and (**b**) H-LSCFP analyzed by FIB-SEM

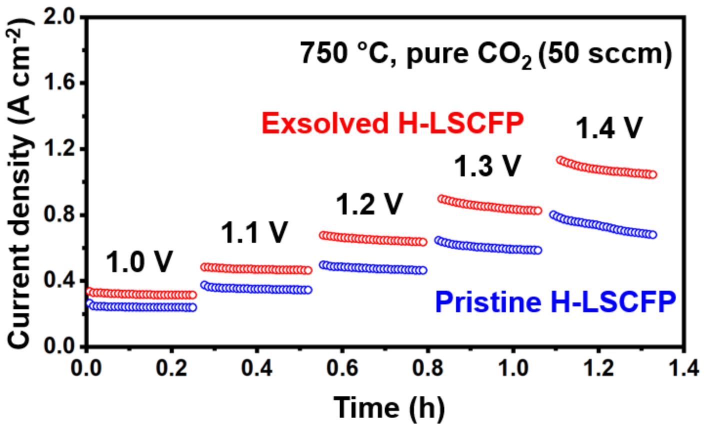

Fig. S15 Short-term potentiostatic tests of the LSGM-supported single cells with H-LSCFP electrode before and after the exsolution treatment conducted in 100% CO_2 of 50 sccm at 750 °C, within a voltage range of 1.0-1.4V

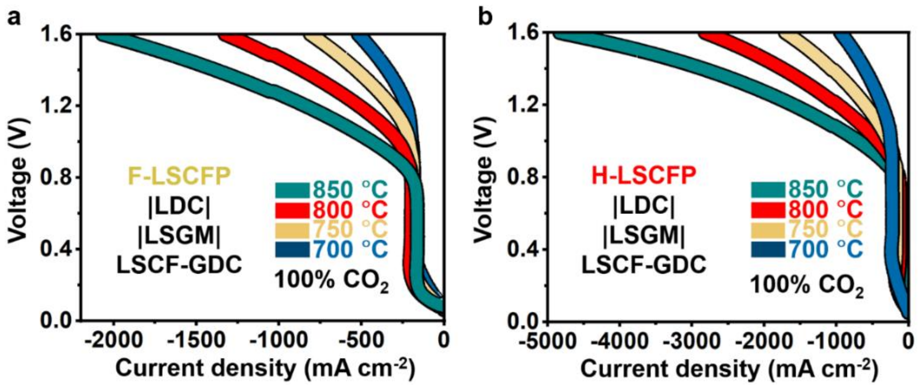

Fig. S16 *I-V* curves of the single cells with the (**a**) F-LSCFP, and (**b**) H-LSCFP fuel electrodes at 700-800 °C in 100% CO_2

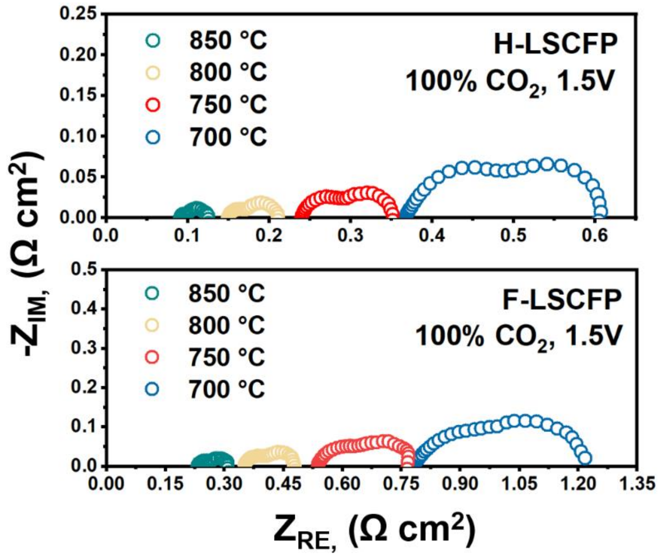

Fig. S17 The Nyquist plots for both H-LSCFP and F-LSCFP cells measured at 700-850 °C, at an applied potential of 1.5 V in 100% CO_2 (50 sccm)

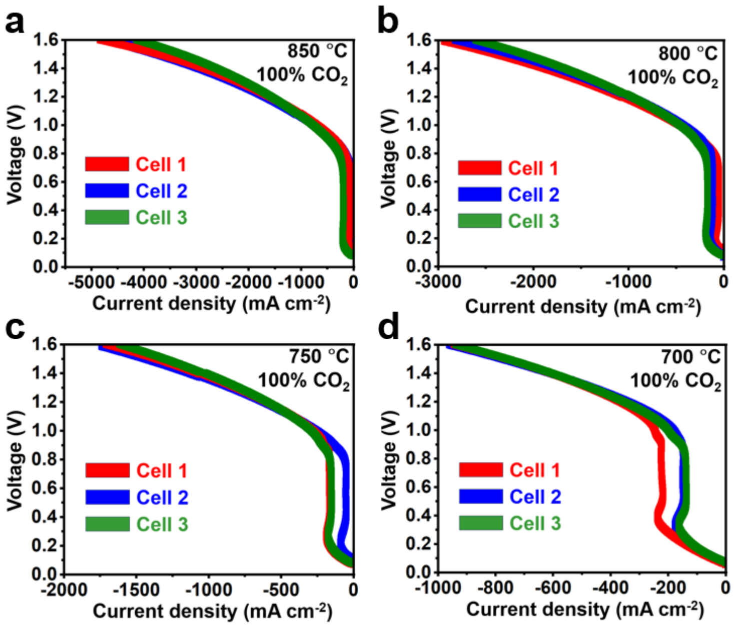

Fig. S18 *I-V* curves of different SOECs with H-LSCFP fuel electrode at **a** 850 °C **b** 800 °C **c** 750 °C **d** 700 °C in 100% CO_2

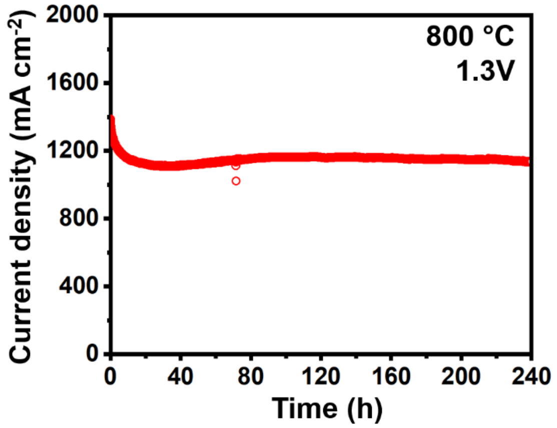

Fig. S19 Stability test of the LSGM-supported single cell with the H-LSCFP-GDC fuel electrode in 100% CO_2 at 800 °C and 1.3 V

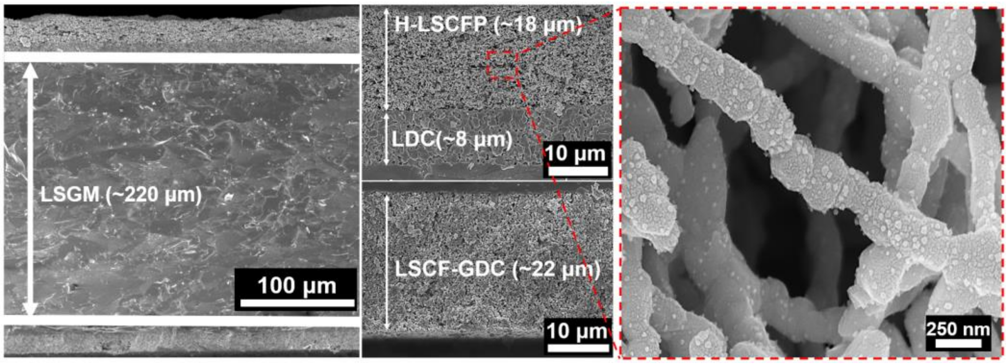

Fig. S20 The cross-sectional SEM image of the LSGM electrolyte-supported H-LSCFP cell after 100h long-term stability test

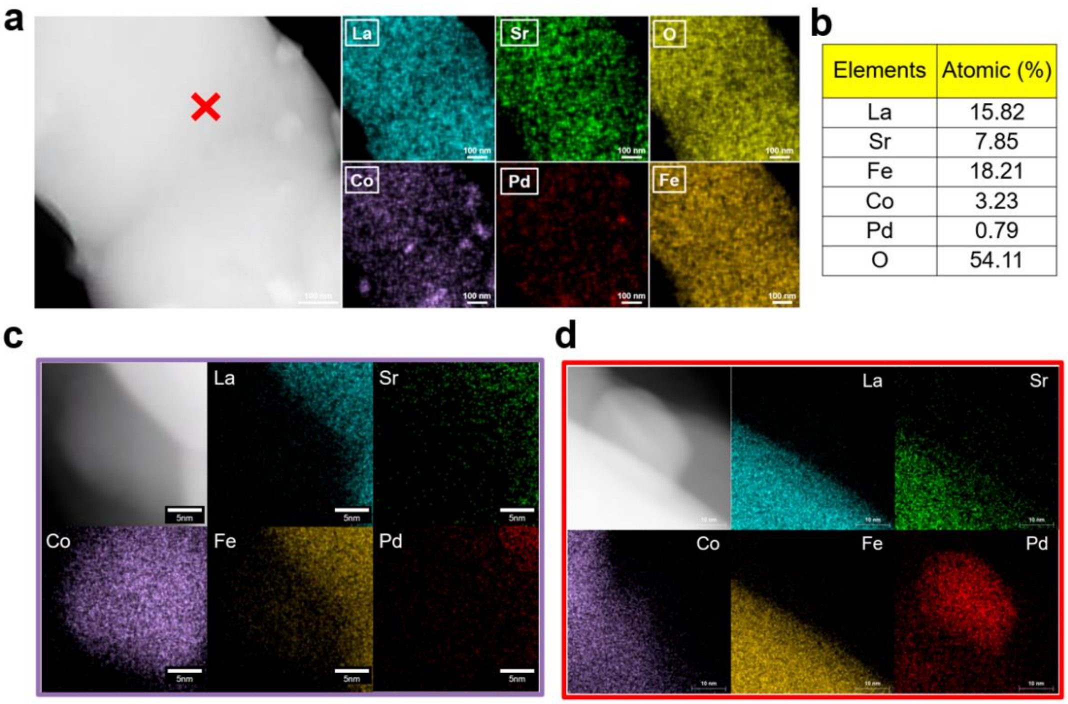

Fig. S21 a TEM image and the corresponding EDX mapping images with **b** point-EDX analyses of the H-LSCFP electrode nanofiber body following the 100-hour long-term test in 100% CO_2 at 800 °C and 1.3 V with the STEM-EDX mappings of exsolved **c** Co and **d** Pd nanoparticles

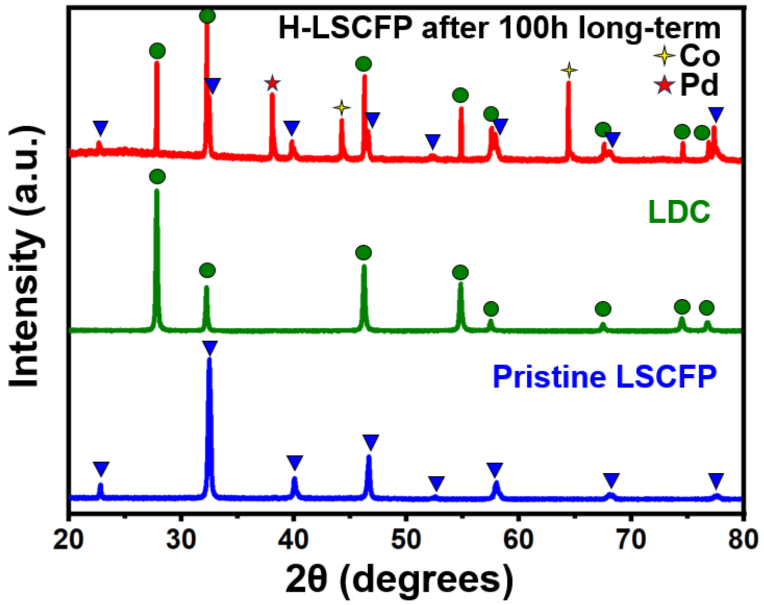

Fig. S22 XRD patterns of the H-LSCFP cell after a 100-h long-term analysis along with pristine LSCFP and LDC patterns

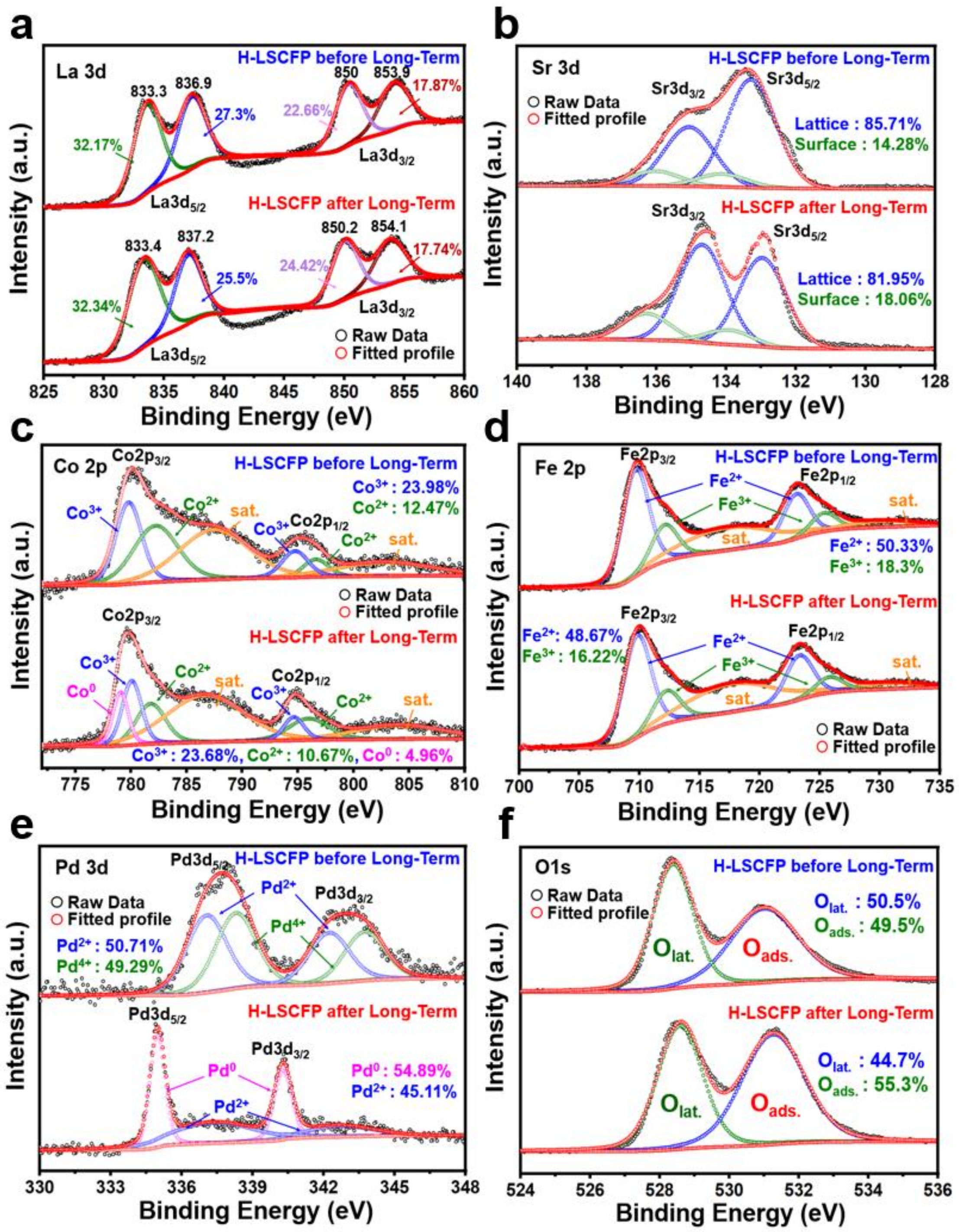

Fig. S23 The oxidation state analysis of the H-LSCFP electrode before and after the 100-h test. High-resolution XPS scans of **a** La 3d, **b** Sr 3d, **c** Co 2p, **d** Fe 2p, **e** Pd 3d, and **f** O1s

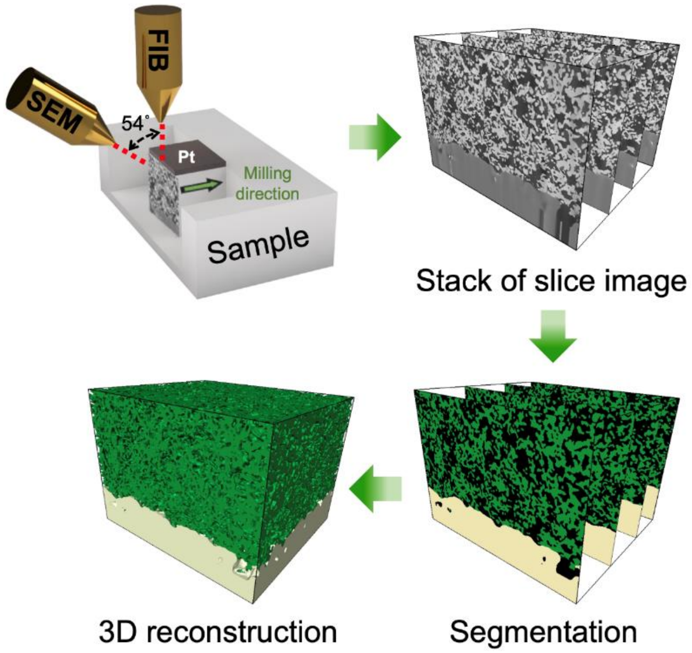

Fig. S24 Schematic diagrams of the 3D reconstruction process

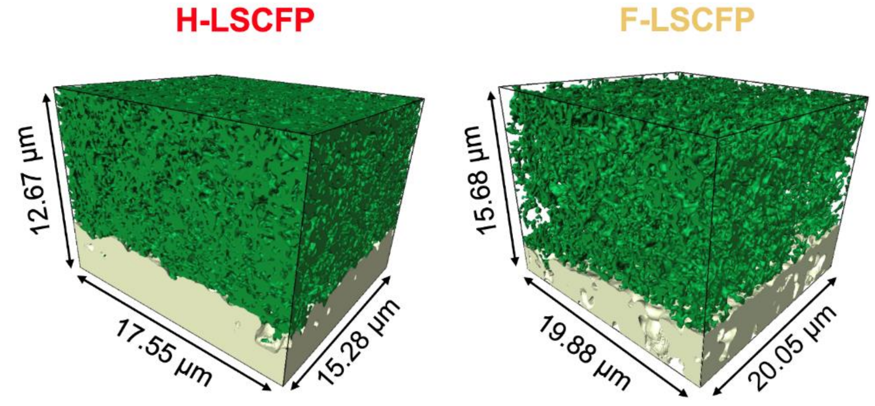

Fig. S25 3D reconstruction of the H-LSCCFP and F-LSCFP electrode samples

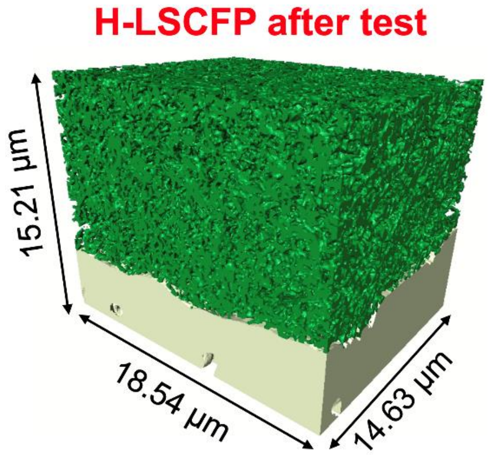

Fig. S26 3D reconstruction of the H-LSCFP electrode after the stability test
